# Spatial and temporal dynamics of ATP synthase from mitochondria toward the cell surface

**DOI:** 10.1038/s42003-023-04785-3

**Published:** 2023-04-18

**Authors:** Yi-Wen Chang, T. Tony Yang, Min-Chun Chen, Y-geh Liaw, Chieh-Fan Yin, Xiu-Qi Lin-Yan, Ting-Yu Huang, Jen-Tzu Hou, Yi-Hsuan Hung, Chia-Lang Hsu, Hsuan-Cheng Huang, Hsueh-Fen Juan

**Affiliations:** 1 Department of Life Science, Institute of Molecular and Cellular Biology, National Taiwan University, Taipei, 106 Taiwan; 2 Department of Electrical Engineering, National Taiwan University, Taipei, 106 Taiwan; 3 Graduate Institute of Biomedical Electronics and Bioinformatics, National Taiwan University, Taipei, 106 Taiwan; 4 Department of Medical Research, National Taiwan University Hospital, Taipei, 100 Taiwan; 5 Institute of Biomedical Informatics, National Yang Ming Chiao Tung University, Taipei, 112 Taiwan; 6 Center for Computational and Systems Biology, National Taiwan University, Taipei, 106 Taiwan

**Keywords:** Systems biology, Cellular imaging, Proteomics

## Abstract

Ectopic ATP synthase complex (eATP synthase), located on cancer cell surface, has been reported to possess catalytic activity that facilitates the generation of ATP in the extracellular environment to establish a suitable microenvironment and to be a potential target for cancer therapy. However, the mechanism of intracellular ATP synthase complex transport remains unclear. Using a combination of spatial proteomics, interaction proteomics, and transcriptomics analyses, we find ATP synthase complex is first assembled in the mitochondria and subsequently delivered to the cell surface along the microtubule via the interplay of dynamin-related protein 1 (DRP1) and kinesin family member 5B (KIF5B). We further demonstrate that the mitochondrial membrane fuses to the plasma membrane in turn to anchor ATP syntheses on the cell surface using super-resolution imaging and real-time fusion assay in live cells. Our results provide a blueprint of eATP synthase trafficking and contribute to the understanding of the dynamics of tumor progression.

## Abstract (web summary)

The ectopic ATP synthase complex is assembled in mitochondria and transported to the cell surface via microtubules and dynamin-related protein 1 and kinesin KIF5B.

## Introduction

Adenosine triphosphate (ATP) synthase, a ubiquitous multimeric protein complex, is an important catalytic enzyme for generating ATP, the common energy currency of cells. This process occurs by facilitating the phosphorylation of adenosine diphosphate by exploiting a transmembrane proton gradient^1^. ATP synthase consists of two discrete domains: the F_O_ domain is embedded within the mitochondrial inner membrane and comprises a proton pore, and the F_1_ domain is located in the mitochondrial matrix and exhibits catalytic activity. The c-ring oligomer of the F_O_ domain rotates when protons flux through it, after which the central stalk of the ATP synthase-γ subunit drives the rotation of the F_1_ domain. The α3β3 subunits of the F_1_ domain proceed with a series of conformational changes, leading to ATP synthesis^2^.

In previous studies, ATP synthase was generally found to be embedded in the inner membrane of mitochondria. However, emerging evidence has shown that several ATP synthase subunits are present on the plasma membrane (PM) of various cell lines, including endothelial cells, adipocytes, keratinocytes, hepatocytes, and some types of cancer cells^3–7^. In addition to cell lines, a pioneering study has recognized the monomeric complex of ATP synthase on the PM of rat liver tissue. The functional activity of this PM translocating ATP synthase complex is modulated in short-term extrahepatic cholestasis by interacting with ecto-ATPase inhibitory factor 1 (ecto-IF1), a mitochondrial regulatory protein ectopically expressed on the plasma membrane rat hepatocytes, as well^8^. Based on its ectopic location, this type of ATP synthase is referred to as ectopic ATP (eATP) synthase. Similar to the ATP synthase located on mitochondria, this cell-surface eATP synthase possesses catalytic activity that facilitates the generation of ATP in the extracellular environment to establish a suitable microenvironment^4,9^. For instance, cancer cells under hypoxic conditions increase the catalytic activity of their eATP synthase to adapt to this unfavorable environment^10–12^. In addition, ATP synthase subunit β (ATP5B), which is located on the PM, is a high-density lipoprotein receptor involved in the regulation of cholesterol homeostasis in HepG2 hepatocellular carcinoma cells^13,14^. eATP synthase is also expressed in antigen-presenting cells to facilitate the transportation of human immunodeficiency virus 1 (HIV-1) to immune cells^15^.

An increasing number of studies have identified eATP synthase as a potential molecular target for cancer therapy^16–18^, with supporting findings indicating that angiostatin, a C-terminal fragment peptide derived from plasminogen by serine proteinase cleavage^19,20^, and antibodies against ATP synthase subunits α and β markedly suppress the enzymatic activity of eATP synthase on the cell surface. As a consequence, there is suppression of proliferation, migration, and angiogenesis in endothelial cells^21,22^. Since then, numerous eATP synthase inhibitors have been investigated. Treatment with these eATP synthase inhibitors reduces the production of extracellular ATP and leads to cytotoxic effects in neurons^23^, adipocytes^24^, and vascular endothelial cells^25,26^. In addition to normal cells, eATP synthase blockade also inhibits the proliferation of various types of tumor cells^3,27,28^. Many studies have revealed that the eATP synthase inhibitor citreoviridin induces cell cycle arrest and suppresses proliferation in both breast and lung cancer cells in vitro and in vivo^17,29–32^.

Although eATP synthase has been studied for many years, the mechanism of its transport to the cell surface remains unclear. Accumulating reports have demonstrated that the subcellular localization of proteins is dynamic and intimately related to their functions due to the diverse surrounding environment and potential interacting partners^33,34^. Due to the substantial advances in mass spectrometry, the spatial proteomic approach, which fractionates the interesting organelles, followed by MS analysis, provides the landscape of protein localization in cells^35,36^. Therefore, in this study, we employed a multi-omics approach, including spatial proteomics, interaction proteomics, and transcriptomics, as well as real-time live-cell imaging to reveal the spatial and temporal dynamics of mitochondrial ATP synthase in cancer cells. Spatial proteomics was utilized to reveal the subcellular distribution of ATP synthase, while transcriptomics was used to identify the biological function, mitochondrial transport along the microtubule, which is involved in eATP synthase trafficking. Additionally, interaction proteomics and protein-protein interaction simulation were employed to identify the crucial proteins regulating the ATP synthase trafficking from mitochondria to the PM. Finally, several molecular and cellular experiments, including gene silencing and protein truncation experiments, super-resolution imaging, immunofluorescence, and flow cytometry, were used to validate the eATP synthase trafficking pathway.

## Results

### ATP synthase complex is assembled in the mitochondria and moves from the perinuclear region to the PM

**Fig. 1:**
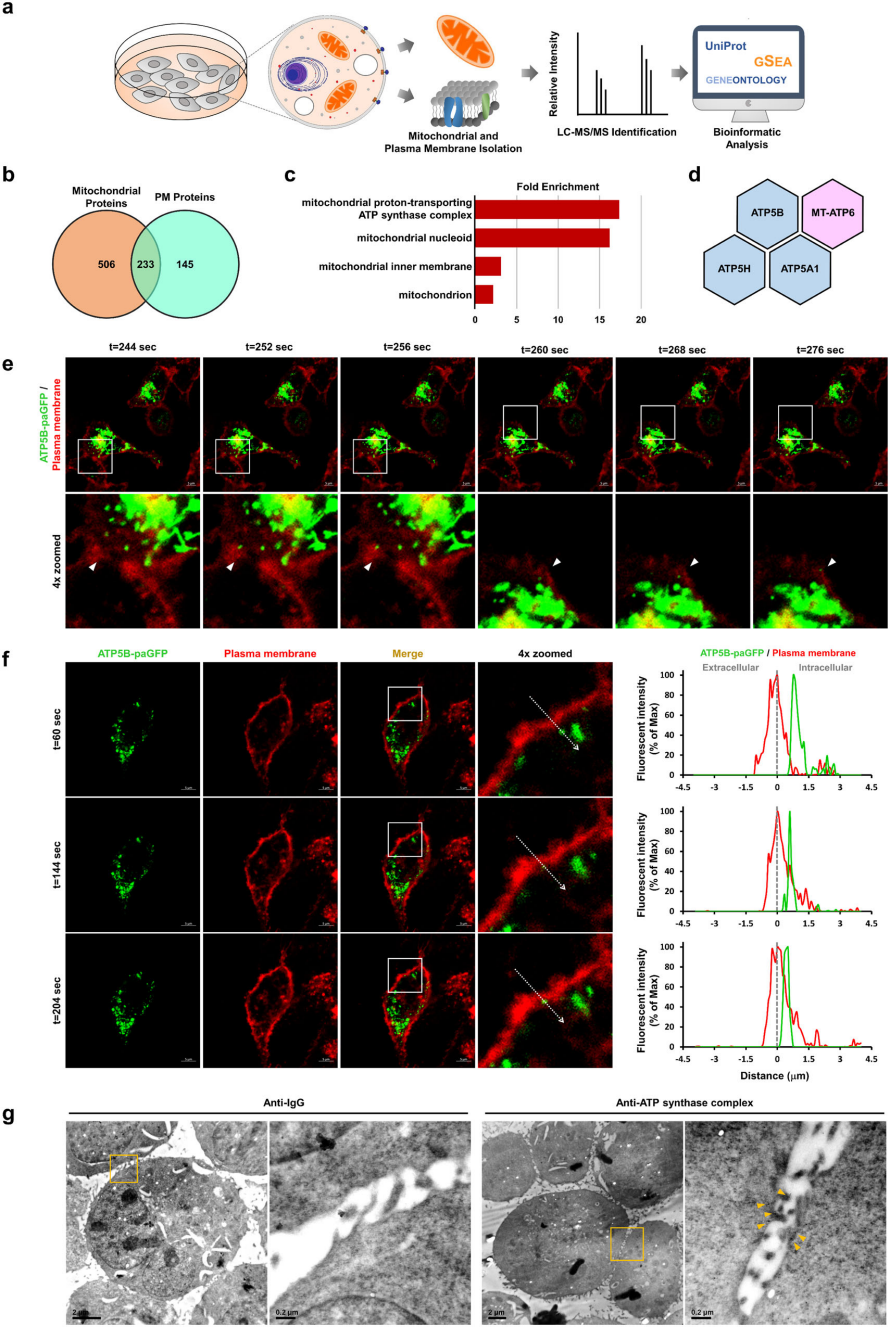
ATP synthase is routed to the cell surface via a mitochondria-dependent trafficking pathway. **a** Schematic representation of the cell surface and mitochondrial proteomes. **b** A venn diagram displays the number of proteins in common between the mitochondria and PM of A549 cells. **c** Functional enrichment analysis of dual-localized proteins in mitochondria and PM. The terms were ranked according to fold enrichment, as shown on the x-axis. **d** The subunits of the ATP synthase complex that are common to both the mitochondria and the PM. The blue color represents the nucleus-encoded subunits, while the pink color represents the mitochondria-encoded subunit. **e** Time-lapse images of ATP5B-paGFP signal (green) in live A549 cells were visualized using confocal microscopy (63× with 2× zoom). The plasma membrane was labeled using CellMask (1:10,000 dilution). Arrows show the colocalization of the ATP5B subunit and the PM. The capture times after photoactivation with a 405 nm laser are shown. Scale bars, 5 µm. **f** Fluorescent signals of real-time tracing images are shown independently or merged. The fluorescence intensity profile for each channel (green and red represent ATP5B-paGFP and the PM, respectively) across the white dashed arrow on the enlarged image is shown in the line graph. The dotted lines indicate the plasma membrane. Scale bars, 5 µm. **g** Immunogold-electron microscopy (immunogold-EM) was used to investigate the localization of ATP synthase in A549 cells. First, ultrathin sections of the cells were stained with an anti-ATP synthase antibody at a dilution of 1:250. Next, a 12 nm gold-conjugated secondary antibody was used to label the ATP synthase protein. The small black dots indicated by arrowheads show the location of the gold-labeled ATP synthase. Mouse IgG, as the negative control, shows the absence of gold particle signals in TEM sections. Scale bars, 2 μm (left) and 0.2 μm (right, zoom-in on the yellow box).

Our previous studies have demonstrated the presence of eATP synthase on the cell surface of fixed cells using flow cytometry and immunofluorescence^29^. Without permeabilization, the antibody can only recognize the cell-surface ATP synthase, which distribution pattern showed a great difference from mitochondrial ATP synthase^17^. Furthermore, the ATP synthase β subunit has also been identified in plasma membrane proteins enriched by biotinylated purification^29^. Additionally, two other mitochondria proteins with differential abundance on the plasma membrane of cells were used as the positive and negative control, respectively. The immunostaining images showed that adenine nucleotide translocase (ANT), another mitochondrial protein known to be reported resident at the PM^37,38^, was detected on the cell surface of non-permeabilized cells (Supplementary Fig. 1a). In contrast, Cyclophilin D, which is located only in mitochondria, was rarely found on the PM (Supplementary Fig. 1b). To further examine whether the whole mitochondrial ATP synthase complex was routed to the PM, we conducted an integrative analysis of the spatial proteomes and identified the proteins located on both mitochondria and the PM (Fig. 1a). Altogether, 739 proteins were identified from purified mitochondria, and 378 were identified in the PM proteome (Fig. 1b and Supplementary Data 1, 2). A comparison of the mitochondrial and PM proteomes revealed 233 proteins that existed in both mitochondria and the PM (Fig. 1b and Supplementary Data 3). Surprisingly, citrate synthase, a key enzyme in the TCA cycle, was also identified in the PM fraction. To verify this interesting finding, immunofluorescence was performed using an antibody against citrate synthase in the non-permeabilized cells (Supplementary Fig. 1c). This finding is consistent with the study of Srere et al., which demonstrated the association of citrate synthase with the inner surface of the mitochondrial inner membrane^39,40^. Furthermore, several reports have observed the binding of other Krebs cycle enzymes located in the mitochondrial matrix with the mitochondrial inner membrane^41,42^. The Gene Ontology (GO) enrichment analysis of these proteins showed that function related to the mitochondrial proton-transporting ATP synthase complex was enriched (Fig. 1c). The ATP synthase complex included ATP5B, ATP synthase subunit a (MT-ATP6), ATP synthase subunit d (ATP5H), and ATP synthase subunit α (ATP5A1) (Fig. 1d). Mitochondria-encoded subunit lacks the membrane-targeting sequence that targets them to the PM of cells. However, its presence on PM implies that the ATP synthase complex may be assembled in the mitochondria and subsequently transported to the cell surface.

Because there is limited real-time evidence regarding the dynamic spatial movement of ATP synthase, we attempted to monitor the subcellular trafficking of ATP synthase in live cells using real-time live imaging. Most fluorescent proteins are easily photobleached, making long-term observation difficult. To extend long-term observation, we manipulated photo-activatable green fluorescent protein (paGFP), which displays a 100-fold higher green fluorescence intensity and is more stable over several days than GFP^43^. After photoactivation, the redistribution of activated paGFP could be followed over time. This allowed us to track the same targeted components that had been activated at the beginning of the experiment, instead of unlabeled, newly-synthesized components. First, we introduced the sequence of ATP5B, an enzymatic β subunit of the ATP synthase complex, into the paGFP vector (Supplementary Fig. 2a). Next, we transfected this recombinant construct into lung cancer cells to observe the transport of ATP synthase. Following stimulation with 405 nm light, the fusion of paGFP with ATP5B (ATP5B-paGFP) was recorded every 0.4 s for 15 min (Supplementary Fig. 2b). Microscopy images revealed that the ATP5B-paGFP signal became detectable after exposure to a pulse of 405 nm light (Supplementary Fig. 2c). Consistently with this, western blotting with antibodies against GFP confirmed that cells transfected with the ATP5B-paGFP construct expressed the ATP5B-paGFP fusion protein (Supplementary Fig. 2d). To identify the subcellular locations of ATP5B-paGFP, MitoTracker Red was used to label mitochondria (Supplementary Fig. 2e). Co-immunoprecipitation was also performed using anti-GFP or anti-ATP5A antibody to confirm that the ATP5B-paGFP fusion protein is assembled in the ATP synthase complex (Supplementary Fig. 2f). Although fluorescence colocalization analysis indicated that approximately 98% of ATP5B-paGFP colocalized with the mitochondria (Supplementary Figs. 2g–i), the time-series images showed that a substantial amount of ATP5B-paGFP was trafficked from the cytosolic region to the PM (Fig. 1e, f and Supplementary Movies 1-4).  The process of ATP synthase trafficking from the cytosol to the PM took from a few seconds to several minutes. Furthermore, the use of a specific immunogold labeling antibody against the ATP synthase complex, in combination with transmission electron microscopy (TEM), known as immunogold-electron microscopy (immunogold-EM), provided high-resolution visualization of the subcellular localization of ATP synthase in cancer cells. Gold-labeled ATP synthase particle was detected not only in the cytosolic region but also in very close proximity to the PM (Fig. 1g). The process of ATP synthase trafficking from the cytosol to the PM took from a few seconds to several minutes. These evidence suggests that eATP synthase is assembled in mitochondria and transported toward the cell surface.

### Mitochondria act as delivery vehicles for the transport of assembled ATP synthase to the PM

**Fig. 2:**
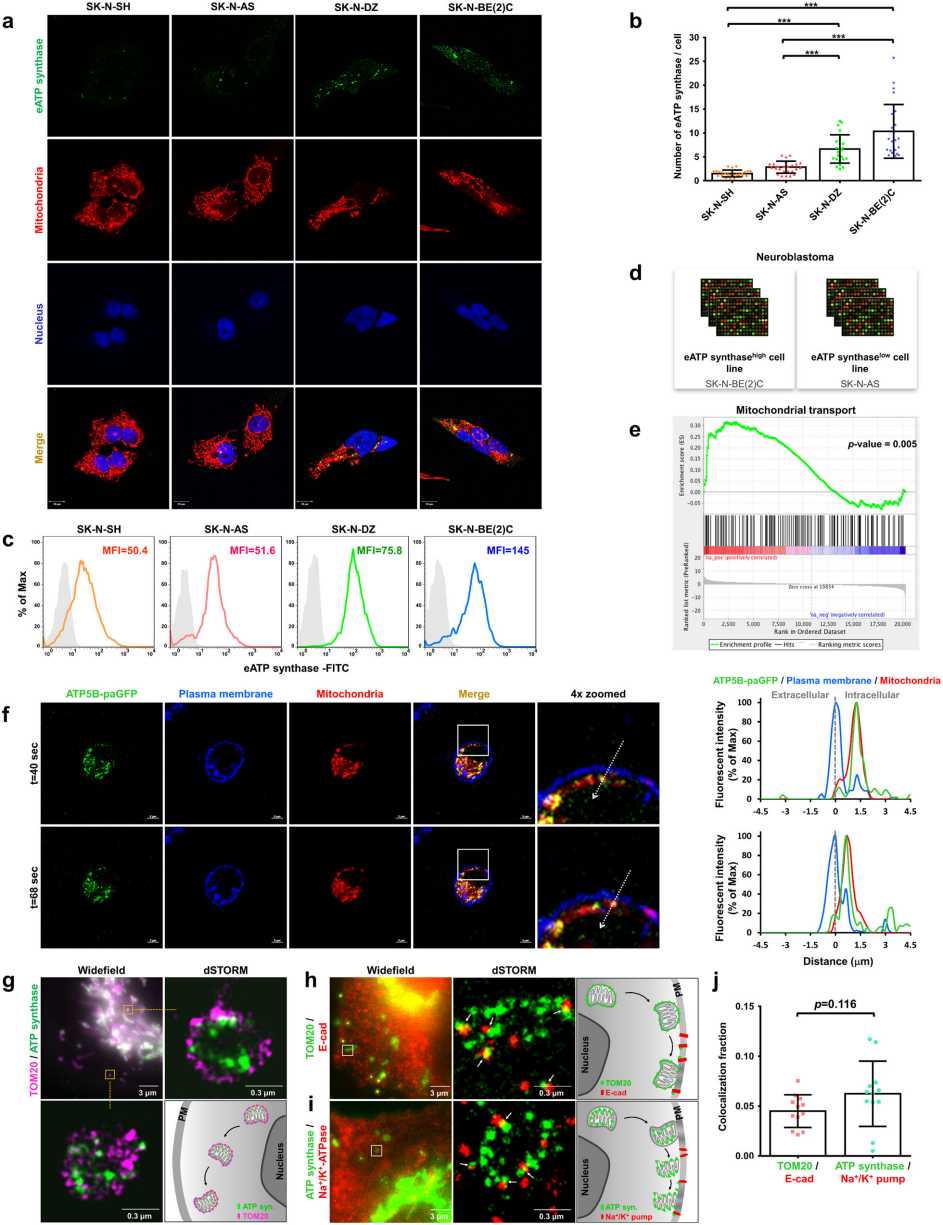
ATP synthase is ectopically translocated on the PM via mitochondria-dependent transport. **a** The abundance of eATP synthase on the cell surface of non-permeabilized neuroblastoma cells was detected using immunofluorescence. The antibody against the ATP synthase complex was used (green). Mitochondria were labeled using MitoTracker (1:10,000 dilution, red), nuclei were labeled using DAPI (blue). Fluorescence signals in the images are shown independently or merged. Scale bars, 10 µm. **b** Quantifications for eATP synthase puncta were measured using ImageJ software. The number of eATP synthase/cell in the y-axis represented the counts of eATP synthase signals on the cell surface per cell. Data presented are mean ± SD (*n* = 20). **c** The expression of eATP synthase on the cell surface was analyzed via flow cytometry, using antibodies for the ATP synthase complex or control mouse IgG. The mean fluorescence intensity (MFI) of eATP synthase based on the flow cytometry data is displayed. **d** The eATP synthase trafficking-related signature was categorized into two groups based on the expression of eATP synthase. **e** Gene set enrichment analysis (GSEA) was performed on the gene expression profiles of the eATP synthase^high^ and eATP synthase^low^ cell lines. The gene sets associated with mitochondrial transport were positively enriched. See also Supplementary Fig. 4. **f** The fluorescence intensity profile for each channel (green, blue, and red represent ATP5B-paGFP, PM, and mitochondria, respectively) across the arrow on real-time tracing images is shown. The dotted lines indicate the plasma membrane. Scale bars, 5 µm. **g** dSTORM images show the distribution of TOM20 (magenta) and ATP synthase complex (green) at mitochondria in cells. **h**, **i** Cells were stained with the antibodies against TOM20 and E-cadherin (**h**) and ATP synthase complex and Na^+/^K^+^-ATPase (**i**). Two-color dSTORM images show the localization between these proteins. **j** The colocalization between the mitochondrial membrane and plasma membrane proteins were further quantified and shown in the bar chart. The values shown are the mean ± SD (*n* = 12). *p < 0.05, **p < 0.01, ***p < 0.001.

In addition to the subunits of ATP synthase, other specific mitochondrial proteins (e.g., mitochondrial import receptor subunit TOM70 (TOMM70), malate dehydrogenase 2 (MDH2), and acetyl-CoA acetyltransferase (ACAT1)) were detected in the PM fractions by proteomic analysis (Supplementary Data 2). We speculated that the eATP synthase probably reaches the PM via mitochondria-dependent transportation. Based on this hypothesis, we performed a bioinformatic analysis to check the correlation between the expression of eATP synthase and mitochondrial trafficking-related genes. First, we measured the expression levels of eATP synthase via immunofluorescence and flow cytometry (Fig. 2a–c and Supplementary Fig. 3a). The presence of eATP synthase, specifically at the basal membrane of neuroblastoma (SK-N-SH, SK-N-AS, SK-N-DZ, and SK-N-BE(2)C cells) and lung cancer (A549 cells), was confirmed using total internal reflection fluorescence (TIRF) microscopy (Supplementary Fig. 3b). Next, we divided neuroblastoma cell lines into two groups based on their expression of eATP synthase. SK-N-BE(2)C and SK-N-DZ cells, which contained more eATP synthase, were defined as eATP synthase^high^ cells, whereas SK-N-AS and SK-N-SH cells were classified as eATP synthase^low^ cells (Fig. 2d). The gene expression profiles of cells representing each group were curated from the Gene Expression Omnibus (GEO) with accession number GSE78061. Using a threshold of *p* < 0.05 and fold change ≥1.5, 496 upregulated and 605 downregulated differential genes were identified in eATP synthase^high^ versus eATP synthase^low^ cells (Supplementary Fig. 4a and Supplementary Data 4). Subsequent functional enrichment analysis showed that many of the differentially expressed genes are involved in the mitochondrial organization and transportation (Supplementary Fig. 4b and Supplementary Data 5). Consistent with this, gene set enrichment analysis (GSEA) revealed that the expression of genes associated with the set-mitochondrial transport (GO: 0006839) was elevated (*p* = 0.005) in eATP synthase^high^ than in eATP synthase^low^ cells (Fig. 2e). In addition to neuroblastoma cell lines, we also found that genes associated with the following GO terms were relatively upregulated in lung cancer cell lines in response to high expression of eATP synthase: Mitochondrion transport along microtubule (GO: 0047497), Establishment of mitochondrion localization, microtubule-mediated (GO: 0034643), and Mitochondrion localization (GO: 0051646) (Supplementary Fig. 4c).

The PM and mitochondria of cells transfected with ATP5B-paGFP were labeled using CellMask Deep Red and MitoTracker Red to observe the relationship between ATP synthase, mitochondria, and the PM. The time-series live-cell images showed that the ATP synthase β subunit was colocalized with mitochondria, and that they moved together toward the PM region (Fig. 2f and Supplementary Movies 5, 6). We further speculated how ATP synthase in the mitochondrial inner membrane transfer to the PM by utilizing dual-color super-resolution microscopy, which provides a nanoscopic resolution to approach molecule dimensions of the proteins. The results showed that signals of TOM20 and the ATP synthase complex simultaneously presented on the mitochondria located near either the nucleus or PM, implying that whole mitochondria, containing the mitochondrial outer membrane (MOM) and the mitochondrial inner membrane (MIM), might be transported toward the cell membrane (Fig. 2g).

**Fig. 3:**
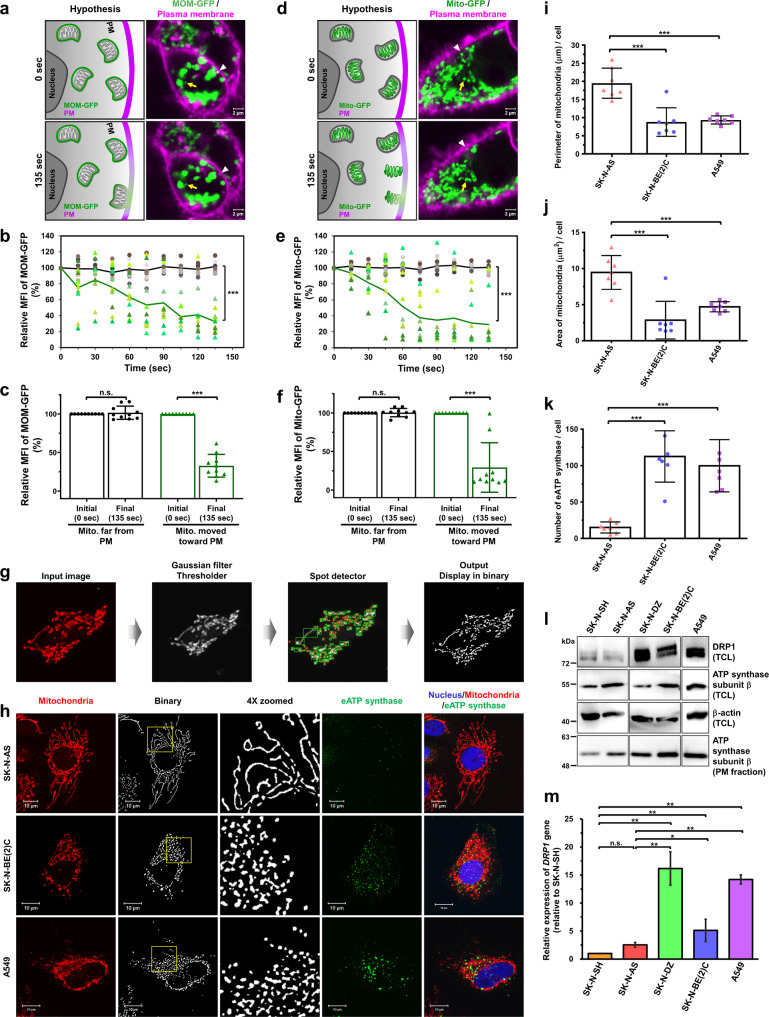
Mitochondrial dynamic morphology influences the expression level of ectopic ATP synthase. **a**–**f** A549 cells were transfected with MOM-GFP or Mito-GFP followed by CellMask Deep Red stain. **a**, **d** Left: schematic of the real-time fusion assay. Mitochondria containing MOM-GFP (**a**) or Mito-GFP (**d**) were traced, and the mean fluorescence intensities (MFIs) of MOM-GFP or Mito-GFP were real-time recorded. The full fusion events were indicated by the diffusion of the MOM-GFP or Mito-GFP signal, which means the redistribution of GFP from mitochondria to PM. Right: MOM-GFP or Mito-GFP signal at 0 and 135 s were shown. The white arrowheads represent the mitochondria that completed mitochondria-PM fusion events, whereas the yellow arrows as unfused mitochondria. **b**, **e** The mean of fluorescence intensities (MFIs) of randomly selected mitochondria in MOM-GFP (**b**) or Mito-GFP (**e**) transfected cells were analyzed by measuring them every 15 s over a period of 135 s and plotted against time. The MFIs at different time points were normalized to 0 s. Each dot represented the relative MFIs of an individual mitochondrion. The triangles filled with green shades represent the relative MFIs of mitochondria that moved toward the proximity of PM (*n* = 10). The green line indicates the average of relative MFIs at the indicated time point. The circles filled with gray shades represent the relative MFIs of mitochondria far from the cell boundary. The black line represents the average of relative MFIs at the indicated time. **c**, **f** The relative MFIs of randomly selected mitochondria in MOM-GFP (**c**) or Mito-GFP (**f**) transfected cells at the initial (0 s) and the final time point (135 s) of the recording process are displayed as bar-dot plots. The values shown are the mean ± SD (*n* = 10). **g** The process of quantifying the mitochondrial area was illustrated using Icy software. **h** The mitochondrial morphology and the expression of eATP synthase in non-permeabilized neuroblastoma and lung cancer cells were visualized using anti-ATP synthase antibody followed by hybridization with Alexa 488 anti-mouse IgG (green) and MitoTracker (red) under confocal microscopy (100×). Nuclei were labeled using DAPI (blue). Binary images were processed using Icy software (lower). Scale bars, 10 μm. **i**–**k** The mitochondrial perimeter (**i**) and area (**j**) were quantified by Icy software, and the count of eATP synthase (**k**) was quantified by ImageJ software (*n* = 7). The number of eATP synthase on the cell surface per cell was shown on the y-axis. The data presented are the mean ± SD. **l** The expression levels of ATP synthase subunit β and DRP1 were detected using western blotting in the total cell lysate (TCL) or PM fraction of various cell lines. **m** The gene expression levels of DRP1 were measured via quantitative RT-PCR in various cell lines. Values were normalized to the expression of GAPDH and relative to the expression of the DRP1 gene in SK-N-SH cells. Values shown are the mean ± SEM (*n* = 3) *p < 0.05, **p < 0.01, ***p < 0.001.

To further investigate the contact between the PM, MOM, and MIM, we respectively labeled these three organelles by using the antibodies against their abundant proteins and observed their relative position via super-resolution imaging. TOM20, a component of the mitochondrial import receptor complex located at the surface of the MOM, contacted with E-cadherin, a transmembrane glycoprotein of PM (Fig. 2h). Also, the ATP synthase complex, an essential catalyst for ATP production located on the MIM, spatial colocalized with the Na^+/^K^+^-ATPase, which is highly expressed on the PM (Fig. 2i). These spatial colocalizations of mitochondrial membrane proteins to PM proteins might result from the fusion of the MOM and PM, and then the MIM and PM. Moreover, the colocalized ratio of TOM20 and E-cadherin has no difference from that of the ATP synthase complex and Na^+/^K^+^-ATPase (Fig. 2j). Based on our results, we were curious about whether TOM20, a MOM protein, was ectopically translocated to the PM, similar to ATP synthase, via the membrane fusion event. Western blotting confirmed that TOM20 was present in the PM fraction, although the expression level of TOM20 was lower than ATP synthase on the cell surface (Supplementary Fig. 1d). In addition to the nanoscale localization of mitochondrial membrane proteins, we designed a real-time tracking system to assess the membrane fusion in live cells. We transfected A549 cancer cells with MOM-GFP plasmid, whose GFP was fused to the mitochondrial outer membrane (MOM) targeting sequence from mitochondrial import receptor subunit TOM70, and labeled the PM of transfected cells using CellMask Deep Red. The time-lapse confocal acquisition of images revealed a partial or complete disappearance of mean fluorescence intensities (MFIs) from GFP-labeled mitochondria that moved to the proximity of the PM. To confirm whether the loss of fluorescence signal resulted from photobleaching, GFP-labeled mitochondria with a similar size to the puncta of interest but located away from the cell boundary served as reference signals. These reference GFP-labeled mitochondria showed only slight MFI changes (Fig. 3a–c). The results indicated that MOM and PM underwent the fusion event that resulted in the diffusion of the MOM-GFP signal (Fig. 3a–c). We also fused GFP to the mitochondrial matrix targeting sequence from subunit VIII of cytochrome c oxidase (Mito-GFP) to detect the MIM-PM fusion in live cells (Fig. 3d–f). Bright-field images of living cells labeled with fluorescence signals were provided to recognize where the nuclei and cell boundaries were (Supplementary Movies 7, 8). TIRF microscopy was carried out to validate the mitochondria-PM fusion events by observing the spread in both MOM-GFP signals (Supplementary Fig. 5a and Supplementary Movie 9) and Mito-GFP signals (Supplementary Fig. 5b and Supplementary Movie 10) at the basal surface of cells. Hence, we suggest that ATP synthase is assembled in mitochondria, then serves as the cargo of microtubule-dependent mitochondrial transport, and finally is transported to the cell surface by attachment of the MOM and MIM with the PM in turn.

### Mitochondrial dynamics change the translocation of ATP synthase to the PM

Recent studies have found that mitochondrial dynamics, such as fusion and fission, is crucial to mitochondrial transport^44^, and increasing the fission rate enhances the efficiency of mitochondrial transport^45^. Therefore, we investigated whether mitochondrial morphology affects the expression of eATP synthase, using the bio-image analysis software Icy^46^ (Fig. 3g). Immunofluorescence revealed that more mitochondrial fission occurred in the eATP synthase^high^ cells A549 and SK-N-BE(2)C compared to the eATP synthase^low^ cells SK-N-AS (Fig. 3h). Quantitative results showed that both the size and perimeter of mitochondria were negatively correlated with the abundance of eATP synthase (Fig. 3i–k). Consistently, the protein and mRNA expression levels of GTPase dynamin-related protein 1 (DRP1), a crucial mediator of mitochondrial fragmentation^47^, were markedly higher in eATP synthase^high^ cells than in eATP synthase^low^ cells (Fig. 3l, m). The expression levels of eATP synthase on the PM fraction of these cell lines were further confirmed using western blotting (Fig. 3l). The results showed that the abundance of eATP synthase was correlated to the level of DRP1. This observation suggests that the transport of eATP synthase may be influenced by the mitochondrial fission/fusion machinery.

### DRP1-dependent mitochondrial fission regulates the anterograde transport of ATP synthase

To reveal the relationship between mitochondrial dynamics and eATP synthase transport. Mitochondrial division inhibitor 1 (Mdivi-1) was used to explore whether the attenuation of mitochondrial fission affects the expression of eATP synthase. The results showed that treatment with Mdivi-1 for 24 h resulted in the elongation of mitochondria (Supplementary Fig. 6a). We then examined whether this affected the abundance of eATP synthase on the cell surface of eATP synthase^high^ cells. Visualized immunofluorescence and flow cytometry indicated that the expression of eATP synthase was downregulated after treatment with Mdivi-1 (Supplementary Figs. 6a–c). The protein levels of Drp1 were reduced after treatment with Mdivi-1 in the eATP synthase^high^ cell lines A549 and SK-N-BE(2)C (Supplementary Fig. 6d), consistent with a previous study that has shown a decrease in both mRNA and proteins levels by Mdivi-1 treatment^48^. The real-time movement imaging of ATP5B-paGFP was recorded to validate the results from immunofluorescence and flow cytometry experiments. Time-series images demonstrated that ATP5B-paGFP moved a shorter distance in Mdivi-1-treated cells than in control cells (Supplementary Fig. 6e, f). Fluorescence colocalization analysis showed that the overlapping proportion of ATP5B-paGFP and Deep Red-labeled PM was reduced in Mdivi-1-treated cells (Supplementary Fig. 6g, h). The previous study has shown that Mdivi-1 treatment induces spindle abnormalities via inhibition of tubulin polymerization in triple-negative breast cancer^49^. Thus, we further validated that Mdivi-1 treatment did not have an inhibitory effect on tubulin polymerization in lung cancer and neuroblastoma (Supplementary Fig. 7). The results help us ensure that DRP1 inhibition plays a role in regulating mitochondrial movement. Taken together, these findings indicate that disruption of mitochondrial dynamics modulates the transport of ATP synthase from the cytosol to the PM.

**Fig. 4:**
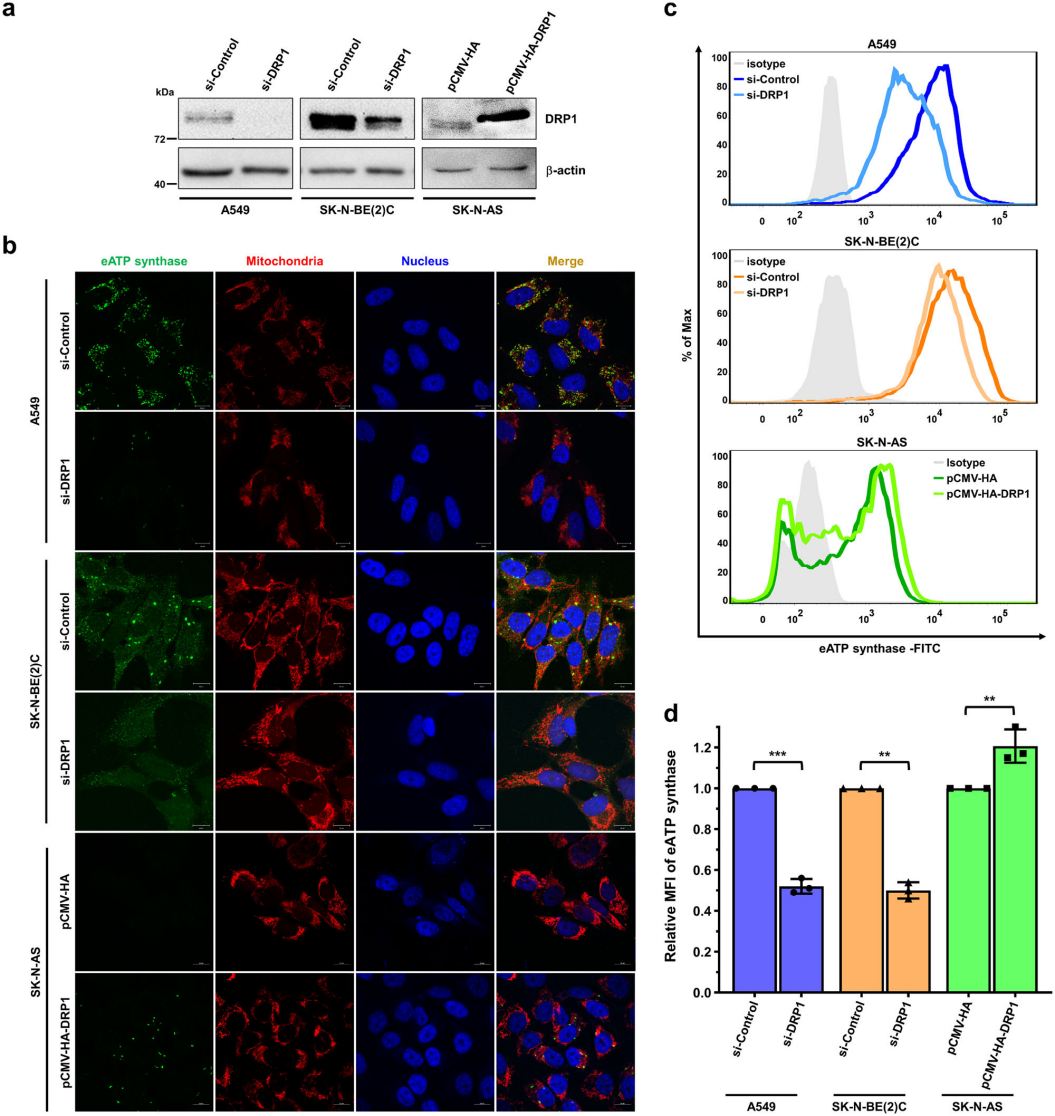
Disturbance of DRP1-dependent mitochondrial dynamics affects the trafficking of ectopic ATP synthase. A549 and SK-N-BE(2)C cells were transfected with siControl or siDRP1, whereas SK-N-AS cells were transfected with the pCMV-HA or pCMV-HA-DRP1 plasmid in **a**–**d**. **a** The knockdown efficiency of siRNA was measured via western blotting using the anti-DRP1 antibody in siControl and siDRP1-transfected A549 and SK-N-BE(2)C cells. The overexpression efficiency was measured using an anti-HA antibody in control and DRP1-overexpressing SK-N-AS cells. **b**–**d** The expression of eATP synthase on the cell surface was detected via immunofluorescence (**b**) and flow cytometry (**c**) using anti-ATP synthase antibody in the knockdown and overexpression cells without permeabilization. The mean fluorescence intensity (MFI) of eATP synthase is displayed as a bar chart (**d**). Values shown are the mean ± SD (*n* = 3). *p < 0.05, **p < 0.01, ***p < 0.001.

In addition, silencing DRP1 was performed in eATP synthase^high^ cells to address the specific regulation of ATP synthase transport by mitochondrial fission. The expression of DRP1 protein in A549 and SK-N-BE(2)C cells transfected with short interfering RNA (siRNA) against DRP1 was severely reduced (Fig. 4a). These DRP1-depleted cells exhibited decreased eATP synthase on the cell surface (Fig. 4b–d). TIRF microscopy was applied to look specifically at the bottom membrane of cells. This technique was helpful in better visualizing the distribution of eATP synthase complex on the PM. Similar to our confocal data, the level of eATP synthase was indeed reduced in DRP1-silencing cells (Supplementary Fig. 8a). Moreover, unlike the downregulation of eATP synthase on the PM, the expression levels of ATP synthase did not change in both cytosolic and mitochondrial fractions after perturbing DRP1 expression (Supplementary Fig. 8b). Our previous findings indicate that eATP synthase on the PM retains its enzyme activity, catalyzing ATP production, and is involved in extracellular ATP accumulation^50,51^. As anticipated, DRP1 knockdown remarkably reduced the extracellular ATP concentration, possibly due to the lower abundance of eATP synthase in the DRP1-silencing cells (Supplementary Fig. 8d). In comparison, in the eATP synthase^low^ cells SK-N-AS, overexpression of DRP1 (Fig. 4a) activated mitochondrial division and elevated the expression of eATP synthase on the cell surface (Fig. 4b–d and Supplementary Fig. 8f).

Combined, these results indicate that DRP1 is involved in the transport of ATP synthase from the cytosol to the PM.

### Microtubules act as the intracellular railroad for ectopic ATP synthase trafficking

**Fig. 5:**
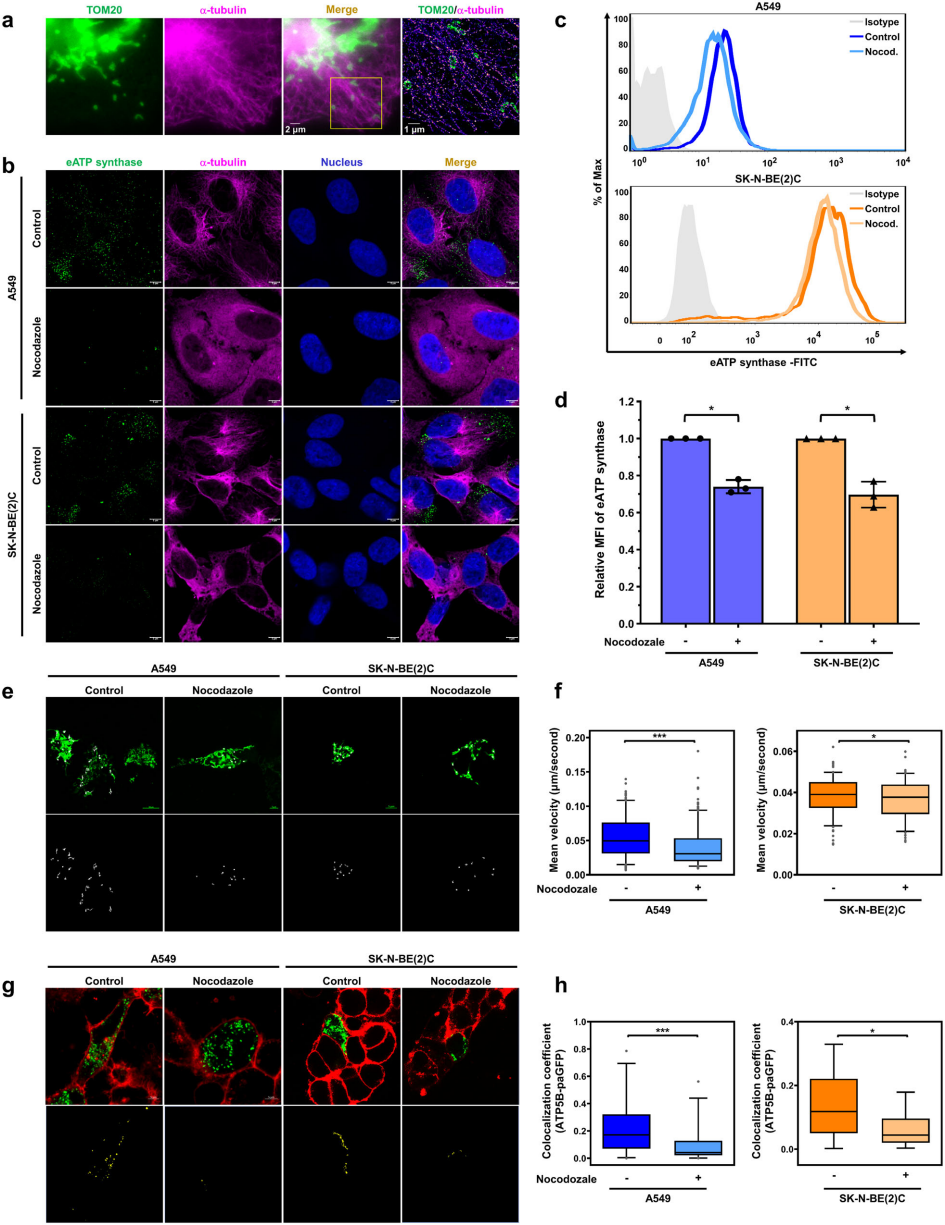
Microtubule-dependent mitochondrial transport regulates the transport of ectopic ATP synthase. **a** Cells were stained with TOM20 (green) and α-tubulin (magenta) to confirm their localization. The 100×oil-immersion objective was used for widefield illumination. The yellow box shows the super-resolution image captured by dSTORM system. Both A549 and SK-N-BE(2)C cells were treated with either DMSO or nocodazole (25 μM) to disrupt their microtubules in **b**–**h**. **b** The expression of eATP synthase on the cell surface was detected using an anti-ATP synthase antibody followed by hybridization with Alexa 488 anti-mouse IgG (green) in non-permeabilized cells. After probing eATP synthase on the cell surface, the cells were subjected to permeabilization, and then the microtubules were labeled using anti-α-tubulin antibody conjugated Alexa 647 (violet). Scale bars, 5 μm. **c** The expression of eATP synthase on the cell surface was detected via flow cytometry using an anti-ATP synthase antibody in the DMSO or nocodazole-treated cells. **d** The bar chart shows the relative MFI of eATP synthase. The data presented are the mean ± SD (*n* = 3). **e** The movement of the ATP5B -paGFP fusion protein in the DMSO and nocodazole groups was recorded for 15 min via confocal microscopy (upper). Their tracks are shown as white lines and were processed using Metamorph software (lower). **f** The dot plot represents the quantification of the translocation velocity in the DMSO and nocodazole groups (*n* = 300 in A549; *n* = 150 in SK-N-BE(2)C). **g** The localization of the ATP5B-paGFP fusion protein (green) and the PM (CellMask; red) was determined using confocal microscopy. The colocalization of these two signals is shown as yellow fluorescence in the merged images (upper), and was further processed using ZEN software (lower). **h** The dot plot shows the colocalization coefficient (colocalization fluorescent area versus total ATP synthase β subunit-paGFP fluorescent area) (*n* = 30 in A549; *n* = 15 in SK-N-BE(2)C). Values shown are the mean ± SD. *p < 0.05, **p < 0.01, ***p < 0.001.

Previous research has shown that in mammalian cells, mitochondrial transport occurs along microtubule tracks^52–56^, which is consistent with the GSEA results of the present study. Therefore, we hypothesized that microtubules may play a critical role in mitochondria-dependent eATP synthase trafficking. We used super-resolution imaging to dissect whether mitochondria localize near microtubules and the results illustrated that the puncta of TOM20, located at the outer membrane of mitochondria, was observed near microtubules (Fig. 5a). To further understand whether microtubules act as the intracellular railroad for ectopic ATP synthase trafficking, nocodazole (a microtubule depolymerization agent) was used to disrupt the microtubules in eATP synthase^high^ cells. After treatment with nocodazole for 1 h, the microtubules were depolymerized and the abundance of eATP synthase was reduced (Fig. 5b and Supplementary Fig. 8c). Flow cytometry also showed that nocodazole inhibited the expression of eATP synthase on the cell surface of both A549 and SK-N-BE(2)C cells (Fig. 5c, d). In addition, time-lapse images revealed that the movement of ATP5B-paGFP was reduced in nocodazole-treated cells versus in control cells (Fig. 5e, f). The colocalization analysis showed that ectopic ATP5B-paGFP, which colocalized with the PM, was also reduced following treatment with nocodazole (Fig. 5g, h). These results illustrate that microtubules are involved in ATP synthase trafficking from the cytosol to the cell surface.

### Kinesin family member 5B (KIF5B) drives the anterograde transport of ATP synthase via the formation of a complex with DRP1

**Fig. 6:**
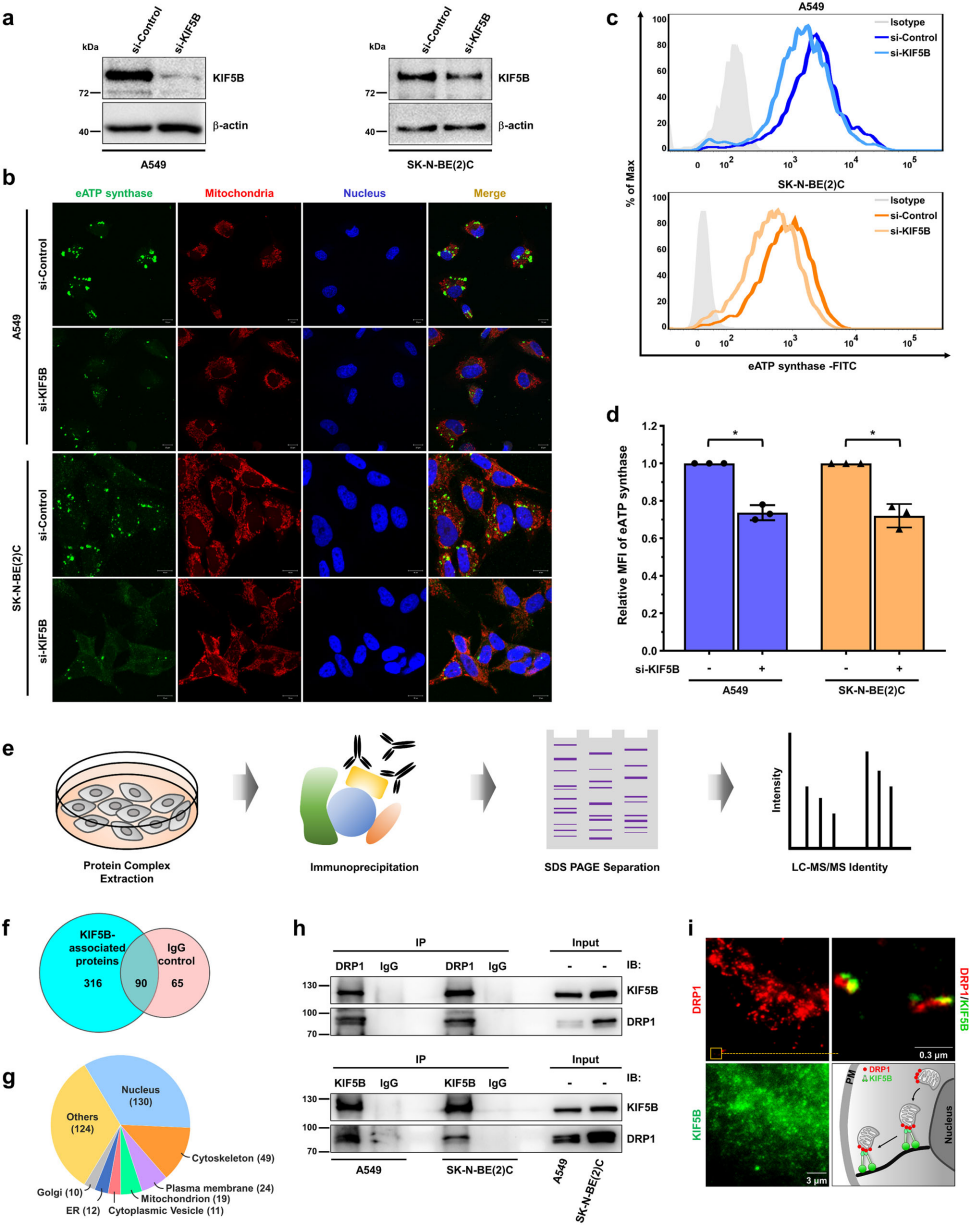
KIF5B and DRP1 form a transport complex to mediate intracellular trafficking of ATP synthase. A549 and SK-N-BE(2)C cells were transfected with siControl or siKIF5B in **a**–**d**. **a** The knockdown efficiency of siRNA was measured via western blotting using an anti-kinesin-1 heavy-chain antibody. **b**–**d** The expression of eATP synthase on the cell surface was detected via immunofluorescence (**b**) and flow cytometry (**c**) using anti-ATP synthase antibody in siControl and siKIF5B-knockdown cells. The relative MFI of eATP synthase is displayed as a bar chart (**d**). The data presented are the mean ± SD (*n* = 3). *p < 0.05, **p < 0.01. ***p < 0.001. **e** Schematic representation of int**e**ractomic profiling using anti-KIF5B or anti-DRP1 antibody. **f** The number of proteins pulled down by the KIF5B-interactome are shown as a Venn diagram. IgG was used as the isotype control of immunoprecipitation. **g** The subcellular localization of KIF5B-interacting proteins. The numbers represent the number of proteins in the indicated subcellular locations. **h** Co-immunoprecipitation with anti-KIF5B antibody or anti-DRP1, followed by western blotting using the indicated antibodies, was performed to verify the interaction between KIF5B and DRP1. Representative images obtained from three independent experiments are shown. **i** Cells were stained with DRP1 (red) and KIF5B (green). The localization of DRP1 and KIF5B were further detected by a dual-color dSTORM imaging system. The diagram shows the relationship between mitochondria, DRP1, and KIF5B.

Kinesin family member 5B (KIF5B, also known as kinesin-1 heavy chain, KINH) is a driver of mitochondrial transport along microtubules^57^. Hence, we examined the relationship between KIF5B and eATP synthase using the siRNA-mediated KIF5B gene knockdown approach. A comparison of siKIF5B and siControl-transfected cells showed that the level of KIF5B protein was lower in the former group (Fig. 6a). Both immunofluorescence microscopy and flow cytometry showed that the abundance of eATP synthase was downregulated in KIF5B-depleted cells (Fig. 6b–d). Due to the less frequent occurrence of eATP synthase trafficking from mitochondria to the cell surface, the extracellular ATP concentration consequently decreased in KIF5B-silencing cells (Supplementary Fig. 8e). Based on these results, we infer that KIF5B is responsible for the transport of eATP synthase.

Nevertheless, the mechanism through which KIF5B interacts with mitochondria remains unclear. KIF5B-interactomic profiling (i.e., immunoprecipitation of KIF5B followed by mass spectrometry analysis) was used to systematically identify KIF5B-interacting proteins to investigate this mechanism (Fig. 6e). A total of 316 proteins were identified in A549 cells (Fig. 6f and Supplementary Data 6) and further classified according to their cellular locations (Fig. 6g and Supplementary Data 7). Nineteen of these proteins were located in mitochondria. Interestingly, DRP1, which affects eATP synthase trafficking via regulation of mitochondrial fission, was identified as one of the KIF5B-interacting proteins. We further validated the interaction between KIF5B and DRP1 in A549 and SK-N-BE(2)C cells by co-immunoprecipitation, using antibodies against KIF5B and DRP1 (Fig. 6h). Dual-color super-resolution imaging further revealed the spatial colocalization of DRP1 and KIF5B (Fig. 6i). As we know, DRP1 is generally localized on the mitochondria, and the results show that DRP1 also localizes around the cell surface with KIF5B. In conclusion, KIF5B plays an essential role in the trafficking of eATP synthase via the formation of a complex with DRP1.

### The variable domain of DRP1 plays a critical role in ectopic ATP synthase trafficking via interacting with KIF5B

**Fig. 7:**
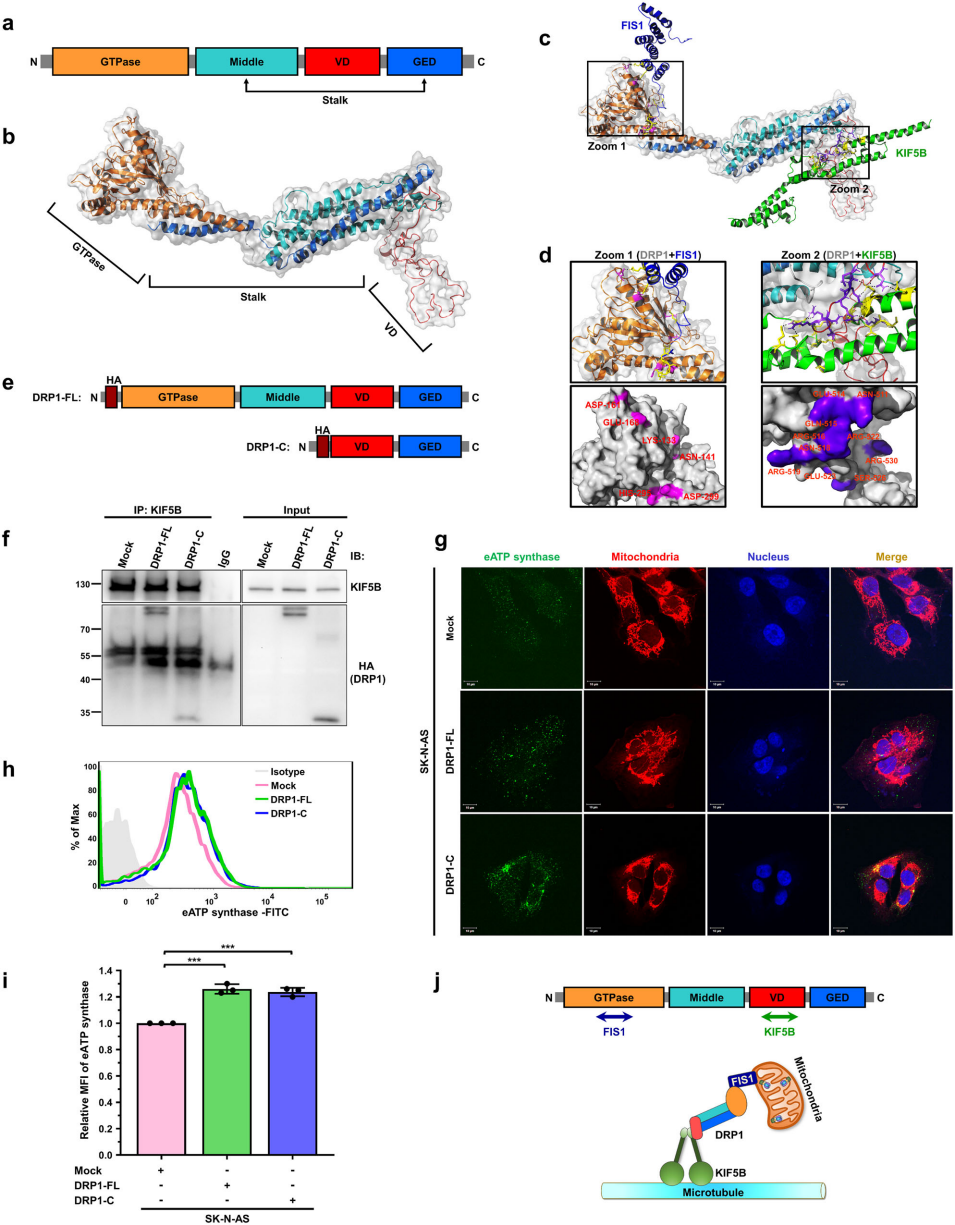
The variable domain of DRP1 is important for the interaction of the DRP1-KIF5B complex regulating the transport of ATP synthase toward the cell surface. **a** Domain organization of DRP1, including the N-terminal GTPase domain (orange), a variable domain (VD, red), and a stalk domain which consists of the middle domain (cyan) and GTPase effector domain (GED, blue). **b** Ribbon and transparent-surface representations show the tertiary structure of DRP1 (PDB ID: 4BEJ) and highlight the conserved domain indicated in the top panel. **c** Overview of FIS1-DRP1 (navy) and KIF5B-DRP1 (green) interfaces. **d** Close-up views of FIS1-DRP1 (Zoom 1) and KIF5B-DRP1 (Zoom 2) interfaces show the two interaction sites, respectively. The residues involved in the interactions are represented as sticks, and hydrogen-bonding interactions are depicted as dashed lines (upper). Surface representation of two interacting sites in DRP1, with purple color indicating KIF5B-interacting sites and violet indicating FIS1-interacting sites. Interface residues are labeled (lower). **e** Scheme of the HA-DRP1 truncated constructs used for co-immunoprecipitation. **f** Co-immunoprecipitation with anti-KIF5B antibody, followed by western blotting using anti-HA antibody, was performed to confirm the interaction between KIF5B and the C-terminal region of DRP1. **g**–**i** The expression of eATP synthase on the cell surface was detected via immunofluorescence (**g**) and flow cytometry (**h**) using anti-ATP synthase antibody in SK-N-AS cells transfected with the indicated DRP1 constructions. The relative MFI of eATP synthase based on the flow cytometry data is displayed as a bar chart (**i**). The data presented are the mean ± SD (*n* = 3). **j** Model for KIF5B-DRP1 complex, which is responsible for the transport of mitochondria. Firstly, DRP1 is recruited to the mitochondria by FIS1, known as a mitochondrial outer membrane protein and interacts with FIS1 with the N-terminal GTPase domain (orange). After mitochondria fission regulation via the FIS1-DRP1 complex, DRP1 leads the fragmented mitochondria to the microtubules by interacting with the motor protein, KIF5B, with variable domain (red). KIF5B-DRP1 complex subsequently acts as a vehicle for mitochondria transportation. *p < 0.05, **p < 0.01. ***p < 0.001.

To further understand how DRP1 interacts with KIF5B, the ClusPro 2.0 web server (https://cluspro.org) was used for protein-protein docking simulation^58^. As shown in Fig. 7a, b, structure-based domain arrangements presented the localization of conserved domains of DRP1 (Protein Data Bank code 4BEJ), including four major domains: the N-terminal GTPase domain, middle assembly domain, variable domain (VD), and C-terminal GTPase effector domain (GED)^59^. Since the crystal structure of the whole KIF5B protein is not available yet, we carried out homology modeling on the full-length sequence of KIF5B obtained from the UniProt database to build a protein structure using SWISS-MODEL^60^. Mitochondrial fission protein 1 (FIS1), which has been reported to recruit DRP1 to mitochondria and mediate mitochondrial fission by forming the FIS1-DRP1 complex^61^, was used as a positive control of the protein-protein docking simulation. The interfaces for KIF5B and for FIS1 were located in two different regions of DRP1 (Fig. 7c). The area for KIF5B-DRP1 interaction containing 10 residues (purple; ASN-511, GLU514, GLN-515, ARG-516, ASN-518, ARG-519, ARG-522, GLU-523, SER-526, and ARG-530) is located on the VD of DRP1 (Fig. 7d; Zoom 2). Interestingly, six residues (violet; LYS-133, ASN-141, ASP-161, GLU-168, HIS-295, and ASP-299), responsible for the interaction of FIS1 and DRP1, are located on the N-terminal GTPase domain of DRP1 (Fig. 7d; Zoom 1). To verify the results of the protein-protein docking simulation, full-length and C-terminal truncated DRP1 constructs (containing VD and GED), which were tagged with HA-tag, were generated (Fig. 7e). Co-immunoprecipitation using antibodies against KIF5B indicated that the C-terminal of DRP1 was sufficient for the interaction of KIF5B with DRP1 (Fig. 7f). Both immunofluorescence and flow cytometry showed that the abundance of eATP synthase was upregulated in SK-N-AS cells overexpressing full-length and C-terminal truncated DRP1 (Fig. 7g–i). These results suggest that DRP1 regulates the trafficking of ectopic ATP synthase via the interaction of its variable domain with KIF5B (Fig. 7j).

## Discussion

Numerous studies have revealed the roles of PM-located ATP synthase (eATP synthase) in cancers^3,5,28^. In this work, we aimed to establish the mechanism responsible for the transport of eATP synthase. We were curious whether the ATP synthase complex was assembled in the mitochondria and then transported to the PM as a whole complex, or whether each subunit of ATP synthase was transported separately and then assembled on the PM. The identification of the mitochondria-encoded subunit, which lacks the PM-targeting sequence, in the PM fraction by spatial proteomic profiling, contributed to our eATP synthase transport. This suggests that ATP synthase subunits, including both mitochondria- and nucleus-encoded subunits, are already assembled in the mitochondria before being transported to the PM. Later, DRP1 promotes mitochondrial fission, which makes mitochondrial movement possible. Moreover, in association with KIF5B, DRP1 carries these fragmented mitochondria containing ATP synthase along the microtubules toward the cell surface. Some evidence points to a positive correlation between eATP synthase and the migratory capability of tumors^3,5^. Identification of these ATP synthase trafficking-related regulators may thus provide useful information for the development of potential therapies against metastatic cancer.

Few studies have explored the question of how ATP synthase reaches the cell surface. To function properly, proteins in eukaryotic cells are usually transported to the appropriate destinations as encoded by short sequences of amino acid peptides, termed signal peptides or targeting sequences. The post-translational translocation pathway transports these proteins directly to the appropriate locations, such as the nucleus, mitochondria, or cytoplasm, via targeting sequences^62^. For instance, proteins with mitochondria-targeting sequences (MTS) are delivered by cytosolic chaperones to the translocase of the outer membrane (TOM) receptors and imported into the mitochondrion by the translocase of the inner membrane (TIM)^63,64^. There are no studies providing evidence regarding the presence of PM-targeting sequences in ATP synthase subunits. It is improbable that these subunits are recruited directly to the PM^10^. In addition, Rai et al. detected eATP synthase on the cell surface of hepatocytes that contained both nucleus- and mitochondria-encoded subunits^65^. Consistent with this, our work verified the presence of mitochondria-encoded subunit a, which lacks the PM-targeting sequence, on the cell surface (Fig. 1d). Moreover, our bioinformatics analysis and real-time live imaging revealed that ATP synthase is transported from the mitochondria to the PM (Fig. 2d–f and Supplementary Fig. 4). This evidence supports our hypothesis that the entire ATP synthase complex is assembled in the mitochondria and then delivered to the cell surface. However, this raises the seminal question of whether there is a possibility of fusion between the mitochondrial membrane and the PM. We used a super-resolution microscope to further reveal whether the mitochondrial membrane attaches to the PM. The results showed that the mitochondrial outer and inner membranes are close to the plasma membrane, in turn to anchor ATP syntheses on the cell surface (Fig. 2g–j). Recent studies have revealed that in budding yeast, a nuclear migration protein (Num1) tethers mitochondria to the inner side of the PM and promotes fusion between these two cellular architectures^66^. Thus, we suggest that in mammalian cells, mitochondria and the PM may fuse via similar machinery, but this hypothesis requires further investigation.

Mitochondrial morphology is constantly changing between the fission and fusion states, and is influenced by metabolic and pathogenic conditions, as these affect the inner and outer environments of the mitochondria^67^. This dynamic state allows mitochondria to communicate with each other to carry out their ordinary functions^68^. Disorders of mitochondrial dynamics lead to a range of disease pathologies^69^. Fusion produces hyper-tubulated forms to enhance communication between interconnected mitochondria. This interconnected mitochondrial network allows mitochondria to complement each other’s deficiencies to promote the health of mitochondria. In contrast, fission creates more fragmented mitochondria, which enhances the oxygen-generation rate and accelerates mitochondrial transport^70^. Accumulating evidence suggests that in cancers, mitochondrial dynamics are imbalanced, with increased fission and decreased fusion^71^. Mitochondrial fission is mediated by DRP1, which oligomerizes to a ring-like structure to facilitate the scission of mitochondria^72^. Recent studies have determined that DRP1-dependent mitochondrial fission is crucial for the specific subcellular localization of mitochondria^59,73,74^. Therefore, based on our transcriptomics analysis and these previous reports, we can infer the relationship between mitochondrial fragmentation and the abundance of ATP synthase. Indeed, our experiments show that a fragmented mitochondrial network enhances the expression of eATP synthase (Fig. 3h–k). Additionally, we disrupted mitochondrial fission by interfering with DRP1 expression (Fig. 4 and Supplementary Fig. 6). The results showed that this disruption triggered mitochondrial fission and affected the abundance of eATP synthase on the cell surface.

Despite the elongation of the mitochondria in lung cancer and neuroblastoma cells treated with Mdivi-1, our additional study indicates that this quinazolinone derivative does not alter mitochondrial morphology and poorly antagonize recombinant DRP1 GTPase activity (Ki >1.2 mM)^75^. Interestingly, Mdivi-1 attenuates mitochondrial ROS by pharmacological inhibition of mitochondrial complex I rather than inhibiting the GTPase activity of DRP1 or elongating mitochondria. These unexpected results suggest that Mdivi-1 is not specific for DRP1 inhibition. Furthermore, Manczak et al. reported the multiple effects of Mdivi-1 on the expression and activities of both electron transport chain (ETC) proteins and mitochondrial dynamics-regulated proteins and networks. Their observations suggest that Mdivi-1 treatment modulates not only the protein level of DRP1 but also other mitochondrial dynamics regulators, including FIS1, MFN1, MFN2, and OPA1. The size and the length of mitochondria increased in Mdivi-1-treated cells, which is consistent with changes in protein expression. Additionally, Mdivi-1 enhances the expression and enzyme activities of ETC proteins^48^. Although Mdivi-1 has been extensively studied, it is important to note the specificity of its effects. Thus, in this study, we verified the role of DRP1 in the intracellular trafficking of eATP synthase using a DRP1 knockdown experiment (Fig. 4). The expression of eATP synthase was reduced and mitochondria were elongated in DRP1-silencing cells, similar to the effects caused by Mdivi-1 (Supplementary Fig. 6). These findings suggest that DRP1-dependent mitochondrial fission is indeed crucial for the trafficking of ATP synthase from mitochondria to the cell surface.

As with most cargos, mitochondrial transport is accomplished by motor proteins, always including kinesin, working along the microtubules by changing their conformation involving ATP hydrolysis^76^. Treatment with microtubule-targeting drugs dramatically inhibits the microtubule-related organelle transport velocity of mitochondria in human neuroblastoma cells^77^. The results of our study demonstrate that disruption of microtubule polymerization suppresses the expression level and translocation velocity of eATP synthase (Fig. 5 and Supplementary Fig. 8c). Generally, kinesin physically attaches itself to the transported cargo via an adapter protein, which acts as a linker between the motor protein and the cargo-surface protein^78^. Tanaka et al. found that, in *kif5B*^*−/−*^ mice, mitochondria were clustered at the minus-ends of microtubules and accumulated around the nucleus in neural cells^79^. In neural cells, a motor complex composed of KIF5B, trafficking kinesin-binding protein 1 (TRAK1) and mitochondrial Rho GTPase (Miro)^72^. Although this model is supported by numerous reports, which suggest that Miro proteins act as adapter sites on the mitochondrial outer membrane for the TRAK/kinesin motor complex and coordinate microtubule-dependent mitochondrial transport^80–82^, recent studies have shown an unexpected observation that approximately 30% of anterograde mitochondrial movement can still occur in cells in the complete absence of Miro proteins^56^. Therefore, other mitochondria-localized proteins, such as mitofusins^83^, MFN2^84^, and Armcx1^85^, may be considered as candidates involved in mitochondrial transport by interacting with the motor complex. These insights can explain our results, which show that inhibiting microtubules or kinesin did not lead to a similar decrease in eATP synthase level as inhibiting DRP1. We cannot exclude the possibility of other potential interactions contributing to mitochondrial transport. In addition to mitochondrial transport, Mx1, a dynamin-related GTPase, is responsible for delivering secretory cargos to the apical or basolateral plasma membrane through interaction with KIF5B^86^. Therefore, we speculate that KIF5B may play a critical role in eATP synthase trafficking by mediating mitochondria-dependent transport. Indeed, our immunofluorescence and flow cytometry analyses showed that silencing KIF5B resulted in reduced eATP synthase abundance (Fig. 6a–d). Combining immunoprecipitation and liquid chromatography–tandem mass spectrometry (IP-LC–MS/MS) spectra, we also found that the mitochondrial outer membrane protein DRP1 associated with KIF5B to transport ATP synthase to the cell surface (Figs. 6e–i, 7). Several studies have reported that kinesin motor proteins are involved in various membrane fission events, such as secretory vesicle formation, endosome morphogenesis, and endocytic vesicle formation^86–88^. Although perturbing KIF5B alone did not affect mitochondrial morphology in our data, it is interesting to investigate whether KIF5B collaborates with DRP1 to facilitate mitochondrial fission.

In conclusion, integrating a multi-omics approach, fluorescence imaging, and bioinformatics analysis help us understand the spatial and temporal dynamics of mitochondrial ATP synthase in cancer cells. In this transport mechanism for eATP synthase, DRP1 leads to mitochondrial fission and subsequently associates with KIF5B to deliver the assembled ATP synthase complex within fragmented mitochondria along microtubules to the cell surface. The mitochondrial outer membrane and inner membrane attach to the plasma membrane, in turn, to anchor ATP syntheses on the cell surface (Supplementary Fig. 9).

## Methods

### Cell cultures

Human lung cancer cell line A549 (ATCC Cat# CCL-185) and neuroblastoma cell lines SK-N-DZ (ATCC Cat# CRL-2149, RRID:CVCL_1701), SK-N-BE(2)C (ATCC Cat# CRL-2268, RRID:CVCL_0529), SK-N-SH (ATCC Cat# HTB-11, RRID:CVCL_0531), and SK-N-AS (ATCC Cat# CRL-2137, RRID:CVCL_1700) were purchased from the American Type Tissue Collection (ATCC). All cell lines were cultured in Dulbecco’s modified Eagle medium (DMEM; Thermo Fisher Scientific Cat# 12800-017) supplemented with 10% fetal bovine serum (FBS; Thermo Fisher Scientific Cat# 10437-028), in a 37 °C incubator with 5% carbon dioxide. Cells were routinely passaged when they reached 80–90% confluency. All cells were detached using trypsin/ethylenediaminetetraacetic acid (trypsin/EDTA; Thermo Fisher Scientific Cat# 15400-054) and transferred to a new culture dish for further incubation. All cell lines were authenticated by matching the STR profile to the ATCC public STR Database.

### Reagents and antibodies

All chemicals used in this study were dissolved in dimethyl sulfoxide (DMSO; Sigma-Aldrich Cat# D2650). Mdivi-1 (Sigma-Aldrich Cat# M0199) was solubilized at 50 mM and nocodazole (Sigma-Aldrich Cat# M1404) was solubilized at 33 mM as stocks. The stocks were stored at −20 °C and freshly diluted in a culture medium at the indicated concentration for treatment. The control samples were treated with an equal volume of DMSO only. The mitochondria in cells were stained with MitoTrackerRed (Thermo Fisher Scientific Cat# M7512) at a 1:10,000 dilution in serum-free medium at 37 °C for 20 min. The PM was stained with CellMask (Thermo Fisher Scientific) at a 1:10,000 dilution in phosphate-buffered saline (PBS) at 37 °C for 10 min. The primary antibodies against the following proteins were used in this study: ATP synthase complex (Abcam Cat# ab109867, 1:1000), KIF5B (Abcam Cat# ab167429, 1:2000), DRP1 (Abcam Cat# ab56788, 1:1000), Na^+^/K^+^-ATPase (Abcam Cat# ab76020, 1:200), TOMM20 (Abcam Cat# ab186735, 1:500), α-tubulin (GeneTex Cat# GTX628802, 1:500), ATP synthase β subunit (GeneTex Cat# GTX84845, 1:1000), HA-tag (BioLegend Cat# 901501, 1:1000), E-cadherin (Merck Millipore Cat# MAB3199Z, 1:200), and Actin (Merck Millipore Cat# MAB1501, 1:5000).

### Experimental design and statistical rationale of multi-omics

For spatial proteomics, PM proteins were extracted from A549 cells using a cell surface isolation kit (Thermo Fisher Scientific Cat#89881), whereas mitochondrial proteins were isolated using the Mitochondria Isolation Kit (Thermo Fisher Scientific Cat#89874) according to the instructions provided by the manufacturer. For immunoprecipitation coupled to mass spectrometry (IP–MS), the total protein of A549 cells was harvested and incubated with the antibody against KIF5B. The interacting proteins of KIF5B were further analyzed via nano-LC-MS/MS. IgG-isotype control was used as the negative control to identify the proteins which had nonspecific binding with Dynabeads Protein A. Each biological sample underwent technical duplicate nano-LC-MS/MS analysis.

### Spatial proteomic profiling

A549 lung cancer cells (2 × 10^7^) were washed twice with PBS. Total proteins from diverse organelles were extracted using a phase-transfer surfactant solution containing 12 mM sodium deoxycholate (Sigma-Aldrich Cat# D6750), 12 mM sodium lauroyl sarcosine (MP Biomedicals Cat# 194009), and 100 mM triethylammonium bicarbonate (TEAB; Sigma-Aldrich Cat# T7408). A protease inhibitor cocktail (BioShop Cat#PIC002) was used to prevent protein degradation. The mixtures were sonicated on ice with a 0.6 s cycle at 60% amplitude and centrifuged at 16,000×*g* for 20 min at 4 °C. The concentration of proteins was measured using an enzyme-linked immunosorbent assay reader (BioRad Model 680) at 570 nm using the Pierce BCA Protein Assay Kit (Thermo Fisher Scientific Cat#23225). A total of 50 μg proteins from each organelle underwent reduction with dithiothreitol/50 mM TEAB at room temperature for 30 min, and alkylation with iodoacetamide/50 mM TEAB at room temperature for 30 min in the dark. Alkylated proteins were digested by Lys-C (1:100 w/w) (WAKO Cat#129-02541) at 37 °C for 3 h and trypsin (1:100 w/w) (Thermo Fisher Scientific Cat#90305) at 37 °C for 16 h. The digested peptides were acidified with trifluoroacetic acid (TFA, Thermo Fisher Scientific Cat# 85183) until the pH was <3, and then treated with ethyl acetate (J.T. Baker Cat# 928001) in 10% TFA to remove detergents. After vacuum-drying using a SpeedVac (EYELA CVE-2100), the peptides were dissolved with 0.1% (v/v) TFA and 5% (v/v) acetonitrile (ACN; Fisher Scientific Cat# A996-4) and subjected to desalting using StageTips with SDB-XC Empore disk membranes (SDB-XC StageTip; 3 M Cat# #2340)^89^.

### Co-immunoprecipitation

After washing twice with PBS, the cells were lysed using Pierce IP lysis buffer (Thermo Fisher Scientific Cat# 87788) with protease inhibitor to repress protein degradation. A total of 3 mg of Dynabeads Protein A (Thermo Fisher Scientific Cat#10002D) were blocked with 5% bovine serum albumin (BioShop Cat#ALB001) at 4 °C for 30 min and incubated with 1 mg cell lysate at 4 °C for 1 h. After precleaning, the precleared cell lysate was further hybridized with 2 μg primary antibody at 4 °C overnight. Mouse or Rabbit IgG (Abcam Cat# ab18457, ab99234) was utilized as the negative control. Protein–antibody complexes were incubated with Dynabeads Protein A at 4 °C for 2 h. The targeted protein-interacting partners were further analyzed by western blotting and nano-LC–MS/MS analysis.

### Nano-LC–MS/MS analysis and proteomic data analysis

The peptides were identified via nano-LC–MS/MS using an LTQ-Orbitrap XL (Thermo Electron, Bremen, Germany) equipped with a nanoACQUITY ultra-performance liquid chromatography system (Waters, Milford, MA)^32^. The raw MS spectra data were analyzed using MaxQuant (RRID:SCR_014485)^90^ for peak detection and protein identification (version 1.3.0.5. for PM proteins; version 1.5.2.8 for mitochondrial proteins and interaction proteomics), using the Swiss-Prot annotated and reviewed database^91^ (published in 2014 for PM proteins, and in 2016 for mitochondrial proteins and interaction proteomics). The parameter settings for the search criteria were identical to those employed in our previous study^32^. The false discovery rate (FDR) threshold of 1% was utilized for protein identification. The identified proteins were analyzed using the Database for Annotation, Visualization, and Integrated Discovery (DAVID) (RRID:SCR_001881) with default settings, and we focused only on the biological process category.

### Gene expression analysis

The gene expression profiles used in this study were mined from Gene Expression Omnibus (GEO) (RRID:SCR_005012) using the R package-GEOquery with accession number GSE78061 for the neuroblastoma cell lines SK-N-DZ, SK-N-BE(2)C, SK-N-AS, and SK-N-SH. According to their expression levels of ectopic ATP synthase determined through flow cytometry, the cell lines were separated into two groups, eATP synthase^high^ and eATP synthase^low^ cells. To avoid the platform effect, we considered only the gene expression profiles provided in Affymetrix UGU 133 plus 2.0, and the raw expression datasets were normalized using the frozen robust multiarray analysis method^92^. The differentially expressed genes were identified using the R package-limma. Genes with *p* < 0.05 and a fold change of ≥1.5 (eATP synthase^high^ versus eATP synthase^low^) were considered differentially expressed genes. The signal-to-noise ratio was calculated to score the gene expression difference between cell lines with high and low abundance of eATP synthase, and the weighted Kolmogorov–Smirnov test was used to identify the regulated gene sets.

### GO enrichment analysis and Gene Set Enrichment Analysis

We used GO-annotated gene sets to interpret the differentially expressed genes. The hypergeometric test was used to identify the over-represented GO terms, as follows:

$$P(x=k)=\mathop{\sum }\limits_{i=k}^{{{\min }}(n,m)}\frac{\left(\begin{array}{c}m\\ i\end{array}\right)\left(\begin{array}{c}N-m\\ n-i\end{array}\right)}{\left(\begin{array}{c}N\\ n\end{array}\right)}$$

*N* is the number of genes in the genome, *m* is the number of genes in the genome that were annotated with a specific GO term, *n* is the number of differentially expressed genes, and *k* is the number of differentially expressed genes that were annotated with a specific GO term. The gene sets were curated from GO using Cytoscape (RRID:SCR_003032)^93^. The gene list, pre-ranked according to fold change, was analyzed using GSEA2-2.2.0^94^.

### Construction and purification of plasmids

The cDNA sequences encoding ATP5B or DRP1 were amplified by PCR using the oligonucleotide primers (Genomics, Hsinchu, Taiwan). The amplified fragments were cloned into a pPAGFP-N1 vector (RRID: Addgene_11909) or pCMV-HA-N vector (Clontech Cat# 635690), respectively. MOM-GFP plasmid containing mitochondrial targeting sequence of Tom70 fused to pEGFP-N1 vector was a gift from Josef Kittler (RRID: Addgene_127633)^95^. Mitochondrial targeting sequence from subunit VIII of human cytochrome C oxidase was amplified from DsRed2-Mito-7, a gift from Michael Davidson (RRID: Addgene_55838) by PCR, and cloned into pEGFP-N1 vector backbone to be Mito-GFP plasmid. All clones were verified by sequencing (Genomics). The *Escherichia coli* DH5α strain was used as the competent cell for the transformation of these constructs. The QIAGEN Plasmid Midi Kit (QIAGEN Cat#12143) was used for the purification of plasmids, and the concentration of the purified plasmids was determined using the NanoDrop ND-1000 (NanoDrop Technologies, Montchanin, DE). Plasmids were transfected into cells using jetPRIME (Polyplus-transfection Cat#114-15) according to the instructions provided by the manufacturer, and the transfected cells were incubated for at least 24 h before further experiments.

### siRNA transfection

The siGENOME SMARTpool siRNAs against KIF5B and DRP1 were obtained from GE Healthcare (Dharmacon Cat# M-008867-00-0020, Dharmacon Cat# M-012092-01-0020), and solubilized with nuclease-free water for further storage. Appropriate quantities of siRNAs were transfected into cells using commercial Lipofectamine 3000 (Invitrogen Cat# L3000015) when the cells reached 60–70% confluency. In brief, the siRNAs and Lipofectamine 3000 reagent were diluted in Opti-Minimal Essential Medium (Thermo Fisher Scientific Cat# 31985070). The diluted siRNAs were mixed with the diluted Lipofectamine 3000 reagent and incubated at room temperature for 10–15 min. Finally, the siRNA–liposome mixture was added to the cells and the medium was replaced after 6 h of transfection to reduce toxicity.

### Western blotting

Proteins were denatured at 95 °C for 5 min, separated by sodium dodecyl sulfate-polyacrylamide gel electrophoresis (SDS-PAGE), and transferred to a polyvinylidene difluoride membrane (PVDF; Merck Millipore Cat# IPVH00010). The PVDF membrane was blocked with 5% milk in Tris-buffered saline with Tween-20 (BioShop Cat# TWN508) (TBST) at room temperature for 1 h. After blocking, the proteins were hybridized with primary antibodies diluted in 5% milk/TBST at 4 °C overnight. The membrane was further incubated with Goat Anti-Mouse IgG H&L (HRP) (Abcam Cat# ab97023) or Goat Anti-Rabbit IgG H&L (HRP) (Abcam Cat# ab97051) diluted with 5% milk/TBST at room temperature for 1 h. The protein bands were analyzed using FluorChem M (ProteinSimple, San Jose, CA).

### Flow cytometry

Cells were detached with 1 mM EDTA (J.T. Baker Cat#8991-01) in PBS at room temperature for 5 min after incubation for at least 24 h. DMEM containing 10% fetal bovine serum was used for the inactivation of EDTA and resuspension of the cells. The resuspended cells were centrifuged at 300×*g* at 4 °C for 5 min and diluted to a concentration of 1 × 10^6^/ml using cold PBS. After fixing with 2% paraformaldehyde at 37 °C in a water bath for 10 min, the non-permeabilized cells were incubated with the primary antibody against ATP synthase at 4 °C overnight. IgG was loaded under the same conditions as an isotype control. Further hybridization utilizing an Alexa 488-conjugated goat anti-mouse or anti-rabbit IgG was performed at room temperature for 1 h. The labeled cells were washed with cold PBS and the signals were detected using a BD FACSCanto II instrument (BD Biosciences, San Jose, CA).

### Real-time live imaging

Cells were seeded into 24-well plates and cultured for 48 h. The ATP5B-paGFP plasmid was transfected using jetPRIME. Prior to the capture of live images, fluorescent probes were used to label the targeted organelles. Images were captured using a Zeiss LSM780 confocal microscope (Zeiss, Oberkochen, Germany) in an incubator at 37 °C and 5% CO_2_. The paGFP signal was activated at a wavelength of 405 nm and detected at 488 nm. Videos were captured every 0.4 s for 15 min to trace the movement of ATP synthase. Acquisition parameters were selected so as to allow the use of minimal laser intensity to prevent photobleaching. The movement distance of the ATP5B-paGFP signal was analyzed using Metamorph software (Molecular Devices, https://www.moleculardevices.com/). This paragraph describes the colocalization analysis between the ATP5B-paGFP signal. The analysis was performed and the PM. The analysis was performed using Zen 2010 software (Zeiss, https://www.zeiss.com/corporate/int/home.html) and involved displaying every pixel in the fluorescence images on a scatterplot based on its fluorescence intensity from each channel. As shown in Supplementary Fig. 2g, the x-axis represented green intensity (ATP5B-paGFP), and the y-axis represented red intensity (Mitochondria). The scatterplot was divided into four quadrants based on negative IgG control and single staining control samples. Quadrant 4 (Q4) represented the pixels with low fluorescent intensities, which were considered background. Q1 represented pixels with green^high^/red^low^ intensities, Q2 represented pixels with green^low^/red^high^ intensities, and Q3 represented pixels with green^high^/red^high^ intensities, which would be considered colocalized signals. The colocalized pixels in Q3 were false-colored to yellow for easier visualization in the confocal fluorescence images (Supplementary Fig. 2h). The green colocalization coefficient of ATP5-paGFP was calculated by summing the pixels in the colocalized region (Q3) and then dividing by the sum of pixels either in the green channel (Q1 + Q3)^96^. The colocalization coefficient values ranged from 0 to 1 (Supplementary Fig. 2i).

### Real-time mitochondria-PM fusion assay

Cells were seeded into 35 mm µ-Dishes and cultured for 24 h. Cells were transfected with MOM-GFP or Mito-GFP plasmid, respectively. After 24 h overexpression, the plasma membrane of cells were labeled using CellMask Deep Red Plasma membrane Stain (Thermo Fisher Scientific Cat#C10046), and the real-time movement of GFP-labeled mitochondria were immediately recorded at 2 frames/sec speed using the Zeiss LSM780 confocal microscope. The Mean fluorescence intensities (MFIs) of selected mitochondria were measured using Metamorph software and normalized to the first image (0 s). Fusion events were identified by changes in MFIs of MOM-GFP or Mito-GFP signal over time.

### Immunofluorescence

Cells were seeded on the coverslips of a 12-well culture plate for 24 h. They were then fixed with 3.7% paraformaldehyde (PFA; Sigma-Aldrich Cat#P6148) in PBS at room temperature for 20 min and washed thrice with PBS. To examine the expression of eATP synthase on the cell surface, the cells were not permeabilized. To detect the intracellular proteins, the cells were subsequently permeabilized using 0.1% triton X-100 (Sigma-Aldrich Cat#T8787) in PBS. Next, the cells were incubated with 10% bovine serum albumin in PBS at room temperature for 1 h to block nonspecific binding. Cells were washed with PBS and hybridized with specific primary antibodies at 4 °C overnight (16–18 h). After removing nonspecific binding by washing with PBS, the cells were incubated with Alexa 488-conjugated Goat anti-mouse IgG (Invitrogen Cat#A11001) or Alexa 488-conjugated Goat anti-rabbit IgG (Invitrogen Cat#A11008) at room temperature for 1 h. Finally, the coverslips were mounted onto the glass slides with 4’,6-diamidino-2-phenylindole (DAPI) containing mounting medium (Invitrogen Cat# P36935). The results were detected using laser scanning confocal microscopy (Zeiss LSM780 with an Airyscan detector and a Plan Apochromat 100 × /1.4 oil objective). The data were adjusted using the Zen 2010 software.

### dSTORM super-resolution imaging and analysis

Samples for super-resolution imaging were prepared according to the protocol of immunofluorescence. During imaging, samples were placed in an imaging chamber (Chamlide magnetic chamber, Live Cell Instrument, Seoul, Korea) and immersed in the imaging buffer containing 50 mM Tris-HCl (Sigma-Aldrich Cat# 648317) and 10 mM NaCl (Sigma-Aldrich Cat# S5886) (TN) buffer at pH 8.0 and an oxygen-scavenging system consisting of 60 mM mercaptoethylamine (Sigma-Aldrich Cat# 641022), pH 8.0, 0.5 mg/ml glucose oxidase (Sigma-Aldrich Cat# G7141), 40 µg/ml catalase (Sigma-Aldrich Cat# C3556), and 10% glucose (Sigma-Aldrich Cat# G8270). The super-resolution imaging were performed on the direct stochastic optical reconstruction microscopy (dSTORM) system, including a modified inverted microscope (Eclipse Ti-E, Nikon, Tokyo, Japan) with a 100 × oil-immersion objective (1.49 NA, CFI Apo TIRF, Nikon)^97,98^. Three light sources: a 647 nm laser (OBIS 637 LX 140 mW, Coherent, Santa Clara, CA, USA), a 561 nm laser (Jive 561 150 mW, Cobolt, Solna, Sweden), and a 405 nm laser (OBIS 405 LX 100 mW, Coherent) were homogenized (Borealis Conditioning Unit, Spectral Applied Research, Toronto, Canada) before focused onto the rear focal plane of the objective. The proteins of interest were initially selected based on their subcellular localization using widefield fluorescence microscopy, and were further analyzed using the dSTORM system. For dual-color imaging, the fluorophores Alexa Fluor 637 and Cy3B were excited using laser lines at 647 and 561 nm, respectively, operating at 3−5 kW/cm^2^. To convert a small fraction of the fluorophores from a quenched state to an excited state, a 405 nm light was used. The single-molecule signals were then captured by an electron-multiplying charge-coupled device (EMCCD) camera (Evolve 512 Delta, Photometrics, Tucson, AZ). A total of 10,000–20,000 frames were acquired for an exposure time of 20 ms. Each single-molecule burst was localized using a MetaMorph Super-resolution Module and cleaned with a Gaussian filter with a radius of 1 pixel.

### Mitochondrial image analysis

We analyzed the captured images using Icy to quantify the perimeter and area of mitochondria^46^. Firstly, we loaded the images into Icy (http://icy.bioimageanalysis.org/), and the weak signals were adjusted to enhance image contrast. Subsequently, images were blurred for the networked mitochondria using a Gaussian filter tool, and displayed to check the results. The filtered results were loaded to the K means thresholder for automatic adjustment, and the binary images were output by inputting the images to the thresholder and label extractor^99^. Next, the processed images were loaded into a spot detector with a parameter setting to three-pixel detection. The results revealed the perimeters, areas, and contour level of every detected spot^100,101^.

### Statistics and reproducibility

All data were presented as the mean ± SD of three experimental replicates and analyzed using Student’s unpaired two-tailed *t*-test. A *p* value of <0.05 denoted statistically significant differences. The sample size used to derive each statistic was provided in the Figure legend.

### Reporting summary

Further information on research design is available in the Nature Portfolio Reporting Summary linked to this article.

## Supplementary information

Supplementary Information

Description of Additional Supplementary Files

Supplementary Data 1

Supplementary Data 2

Supplementary Data 3

Supplementary Data 4

Supplementary Data 5

Supplementary Data 6

Supplementary Data 7

Supplementary Data 8

Supplementary Movie 1

Supplementary Movie 2

Supplementary Movie 3

Supplementary Movie 4

Supplementary Movie 5

Supplementary Movie 6

Supplementary Movie 7

Supplementary Movie 8

Supplementary Movie 9

Supplementary Movie 10

Reporting Summary

## Data Availability

All original mass spectrometry data have been deposited to the ProteomeXchange Consortium via the PRIDE partner repository^102^ with the dataset identifiers PXD006791 (plasma membrane proteome), and PXD007036 (mitochondrial proteome). Uncropped and unedited blot images of the main figures are provided in Supplementary Fig. 10. Source data are available in Supplementary Data 8. Other data supporting our study are available from the corresponding author on reasonable request.

## References

[1] Cross RL (1994). Enzyme structure. Our primary source of ATP. Nature.

[2] Pu J, Karplus M (2008). How subunit coupling produces the γ-subunit rotary motion in F1-ATPase. Proc. Natl Acad. Sci. USA.

[3] Li W (2017). Ectopic expression of the ATP synthase β subunit on the membrane of PC-3M cells supports its potential role in prostate cancer metastasis. Int. J. Oncol.

[4] Panfoli I (2020). Potential role of endothelial cell surface ectopic redox complexes in COVID-19 disease pathogenesis. Clin. Med..

[5] Speransky S (2019). A novel RNA aptamer identifies plasma membrane ATP synthase beta subunit as an early marker and therapeutic target in aggressive cancer. Breast Cancer Res. Treat.

[6] Moser TL (1999). Angiostatin binds ATP synthase on the surface of human endothelial cells. Proc. Natl Acad. Sci. USA.

[7] Liu WJ, Chang YS, Chen PY, Wu SP (2021). F1 ATP synthase β subunit is a putative receptor involved in white spot syndrome virus infection in shrimp by binding with viral envelope proteins VP51B and VP150. Dev. Comp. Immunol..

[8] Giorgio V (2010). The ectopic F(O)F(1) ATP synthase of rat liver is modulated in acute cholestasis by the inhibitor protein IF1. J. Bioenerg. Biomembr..

[9] Comelli M, Domenis R, Buso A, Mavelli I (2016). F1FO ATP synthase is expressed at the surface of embryonic rat heart-derived H9c2 cells and is affected by cardiac-like differentiation. J. Cell. Biochem..

[10] Ma Z (2010). Mitochondrial F1Fo-ATP synthase translocates to cell surface in hepatocytes and has high activity in tumor-like acidic and hypoxic environment. Acta Biochim. Biophys. Sin..

[11] Taurino F (2016). Function and expression study uncovered hepatocyte plasma membrane ecto-ATP synthase as a novel player in liver regeneration. Biochem. J.

[12] Li Y (2021). Flagellar hook protein FlgE induces microvascular hyperpermeability via ectopic ATP synthase β on endothelial surface. Front. Cell. Infect. Microbiol..

[13] Martinez LO (2003). Ectopic β-chain of ATP synthase is an apolipoprotein AI receptor in hepatic HDL endocytosis. Nature.

[14] Kita T, Arakaki N (2015). Contribution of extracellular ATP on the cell-surface F1F0-ATP synthase-mediated intracellular triacylglycerol accumulation. Biomed. Res..

[15] Yavlovich A (2012). Ectopic ATP synthase facilitates transfer of HIV-1 from antigen-presenting cells to CD4(+) target cells. Blood.

[16] Fliedner SM (2015). Potential therapeutic target for malignant paragangliomas: ATP synthase on the surface of paraganglioma cells. Am. J. Cancer Res..

[17] Chang YW (2020). Multiomics reveals ectopic ATP synthase blockade induces cancer cell death via a lncRNA-mediated phospho-signaling network. Mol. Cell. Proteomics.

[18] Wang T, Ma F, Qian HL (2021). Defueling the cancer: ATP synthase as an emerging target in cancer therapy. Mol. Ther. Oncolytics.

[19] O’Reilly MS (1994). Angiostatin: a novel angiogenesis inhibitor that mediates the suppression of metastases by a Lewis lung carcinoma. Cell.

[20] Gately S (1996). Human prostate carcinoma cells express enzymatic activity that converts human plasminogen to the angiogenesis inhibitor, angiostatin. Cancer Res..

[21] Moser TL (2001). Endothelial cell surface F1-FO ATP synthase is active in ATP synthesis and is inhibited by angiostatin. Proc. Natl Acad. Sci. USA.

[22] Yamamoto K (2007). Involvement of cell surface ATP synthase in flow-induced ATP release by vascular endothelial cells. Am. J. Physiol. Heart Circ. Physiol..

[23] Ravera S (2011). Evidence for ectopic aerobic ATP production on C6 glioma cell plasma membrane. Cell. Mol. Neurobiol..

[24] Arakaki N, Kita T, Shibata H, Higuti T (2007). Cell-surface H+-ATP synthase as a potential molecular target for anti-obesity drugs. FEBS Lett.

[25] Alard JE (2011). Autoantibodies to endothelial cell surface ATP synthase, the endogenous receptor for hsp60, might play a pathogenic role in vasculatides. PLoS ONE.

[26] González-Pecchi V (2015). Apolipoprotein A-I enhances proliferation of human endothelial progenitor cells and promotes angiogenesis through the cell surface ATP synthase. Microvasc. Res..

[27] Park BN (2019). Zr-89 immuno-PET targeting ectopic ATP synthase enables in-vivo imaging of tumor angiogenesis. Int. J. Mol. Sci..

[28] Lu ZJ (2009). Identification of ATP synthase beta subunit (ATPB) on the cell surface as a non-small cell lung cancer (NSCLC) associated antigen. BMC Cancer.

[29] Chang HY (2012). Ectopic ATP synthase blockade suppresses lung adenocarcinoma growth by activating the unfolded protein response. Cancer Res..

[30] Chang HY, Huang TC, Chen NN, Huang HC, Juan HF (2014). Combination therapy targeting ectopic ATP synthase and 26S proteasome induces ER stress in breast cancer cells. Cell Death Dis..

[31] Wu YH (2013). Quantitative proteomic analysis of human lung tumor xenografts treated with the ectopic ATP synthase inhibitor citreoviridin. PLoS ONE.

[32] Hu CW (2015). Temporal phosphoproteome dynamics induced by an ATP synthase inhibitor citreoviridin. Mol. Cell. Proteomics.

[33] Bauer NC, Doetsch PW, Corbett AH (2015). Mechanisms regulating protein localization. Traffic.

[34] Guardia CM, De Pace R, Mattera R, Bonifacino JS (2018). Neuronal functions of adaptor complexes involved in protein sorting. Curr. Opin. Neurobiol..

[35] Takamori S (2006). Molecular anatomy of a trafficking organelle. Cell.

[36] Tharkeshwar AK, Gevaert K, Annaert W (2018). Organellar omics-A reviving strategy to untangle the biomolecular complexity of the cell. Proteomics.

[37] Cardouat G (2017). Ectopic adenine nucleotide translocase activity controls extracellular ADP levels and regulates the F(1)-ATPase-mediated HDL endocytosis pathway on hepatocytes. Biochim. Biophys. Acta Mol. Cell Biol. Lipids.

[38] Kliment CR (2021). Adenine nucleotide translocase regulates airway epithelial metabolism, surface hydration and ciliary function. J. Cell Sci..

[39] D’Souza SF, Srere PA (1983). Binding of citrate synthase to mitochondrial inner membranes. J. Biol. Chem..

[40] Moore GE, Gadol SM, Robinson JB, Srere PA (1984). Binding of citrate synthase and malate dehydrogenase to mitochondrial inner membranes: tissue distribution and metabolite effects. Biochem. Biophys. Res. Commun..

[41] Elduque A, Casadó F, Cortés A, Bozal J (1982). Intramitochondrial location of the molecular forms of chicken liver mitochondrial malate dehydrogenase. Int. J. Biochem..

[42] Comte J, Gautheron DC (1978). The markers of pig heart mitochondrial sub-fractions. II. - On the association of malate dehydrogenase with inner membrane. Biochimie.

[43] Karbowski M, Cleland MM, Roelofs BA (2014). Photoactivatable green fluorescent protein-based visualization and quantification of mitochondrial fusion and mitochondrial network complexity in living cells. Methods Enzymol..

[44] Chen H, Chan DC (2009). Mitochondrial dynamics–fusion, fission, movement, and mitophagy–in neurodegenerative diseases. Hum. Mol. Genet..

[45] van der Bliek AM, Shen Q, Kawajiri S (2013). Mechanisms of mitochondrial fission and fusion. Cold Spring Harb. Perspect. Biol..

[46] de Chaumont F (2012). Icy: an open bioimage informatics platform for extended reproducible research. Nat. Methods.

[47] Palmer CS (2013). Adaptor proteins MiD49 and MiD51 can act independently of Mff and Fis1 in Drp1 recruitment and are specific for mitochondrial fission. J. Biol. Chem..

[48] Manczak M, Kandimalla R, Yin X, Reddy PH (2019). Mitochondrial division inhibitor 1 reduces dynamin-related protein 1 and mitochondrial fission activity. Hum. Mol. Genet..

[49] Fang CT, Kuo HH, Yuan CJ, Yao JS, Yih LH (2021). Mdivi-1 induces spindle abnormalities and augments taxol cytotoxicity in MDA-MB-231 cells. Cell Death Discov..

[50] Chang YW (2020). Multiomics reveals ectopic ATP synthase blockade induces cancer cell death via a lncRNA-mediated phospho-signaling network. Mol. Cell. Proteomics.

[51] Chang YW (2022). Quantitative phosphoproteomics reveals ectopic ATP synthase on mesenchymal stem cells to promote tumor progression via ERK/c-Fos pathway activation. Mol. Cell. Proteomics.

[52] Gotoh H, Takenaka T, Horie H, Hiramoto Y (1985). Organelle motility in rat pituitary clonal cells. I. Dynamic movements of intracellular organelles. Cell Struct. Funct..

[53] Morris RL, Hollenbeck PJ (1995). Axonal transport of mitochondria along microtubules and F-actin in living vertebrate neurons. J. Cell Biol..

[54] Chada SR, Hollenbeck PJ (2003). Mitochondrial movement and positioning in axons: the role of growth factor signaling. J. Exp. Biol..

[55] Quintero OA (2009). Human Myo19 is a novel myosin that associates with mitochondria. Curr. Biol..

[56] López‐Doménech G (2018). Miro proteins coordinate microtubule‐and actin‐dependent mitochondrial transport and distribution. EMBO J.

[57] Reis K, Fransson A, Aspenstrom P (2009). The Miro GTPases: at the heart of the mitochondrial transport machinery. FEBS Lett..

[58] Kozakov D (2017). The ClusPro web server for protein-protein docking. Nat. Protoc..

[59] Adachi Y (2016). Coincident phosphatidic acid interaction restrains Drp1 in mitochondrial division. Mol. Cell.

[60] Waterhouse A (2018). SWISS-MODEL: homology modelling of protein structures and complexes. Nucleic Acids Res..

[61] Chen H, Chan DC (2005). Emerging functions of mammalian mitochondrial fusion and fission. Hum. Mol. Genet..

[62] Cokol M, Nair R, Rost B (2000). Finding nuclear localization signals. EMBO Rep..

[63] Gabriel K, Egan B, Lithgow T (2003). Tom40, the import channel of the mitochondrial outer membrane, plays an active role in sorting imported proteins. EMBO J..

[64] Chacinska A, Koehler CM, Milenkovic D, Lithgow T, Pfanner N (2009). Importing mitochondrial proteins: machineries and mechanisms. Cell.

[65] Rai AK, Spolaore B, Harris DA, Dabbeni-Sala F, Lippe G (2013). Ectopic F0F 1 ATP synthase contains both nuclear and mitochondrially-encoded subunits. J. Bioenerg. Biomembr..

[66] Ping HA, Kraft LM, Chen W, Nilles AE, Lackner LL (2016). Num1 anchors mitochondria to the plasma membrane via two domains with different lipid binding specificities. J. Cell Biol..

[67] Wikstrom JD (2014). Hormone-induced mitochondrial fission is utilized by brown adipocytes as an amplification pathway for energy expenditure. EMBO J..

[68] Chan DC (2006). Mitochondrial fusion and fission in mammals. Annu. Rev. Cell Dev. Biol..

[69] Archer SL (2013). Mitochondrial dynamics–mitochondrial fission and fusion in human diseases. N. Engl. J. Med..

[70] Youle RJ, van der Bliek AM (2012). Mitochondrial fission, fusion, and stress. Science.

[71] Caino MC (2016). A neuronal network of mitochondrial dynamics regulates metastasis. Nat. Commun..

[72] Mishra P, Chan DC (2014). Mitochondrial dynamics and inheritance during cell division, development and disease. Nat. Rev. Mol. Cell Biol..

[73] Ishihara N (2009). Mitochondrial fission factor Drp1 is essential for embryonic development and synapse formation in mice. Nat. Cell Biol..

[74] Favaro G (2019). Drp1-mediated mitochondrial shape controls calcium homeostasis and muscle mass. Nat. Commun..

[75] Bordt EA (2017). The putative Drp1 inhibitor mdivi-1 is a reversible mitochondrial complex I inhibitor that modulates reactive oxygen species. Dev. Cell.

[76] Cai Q, Tammineni P (2016). Alterations in mitochondrial quality control in Alzheimer’s disease. Front. Cell. Neurosci..

[77] Smith JA (2016). Structural basis for induction of peripheral neuropathy by microtubule-targeting cancer drugs. Cancer Res..

[78] Isojima H, Iino R, Niitani Y, Noji H, Tomishige M (2016). Direct observation of intermediate states during the stepping motion of kinesin-1. Nat. Chem. Biol..

[79] Tanaka Y (1998). Targeted disruption of mouse conventional kinesin heavy chain kif5B, results in abnormal perinuclear clustering of mitochondria. Cell.

[80] Guo X (2005). The GTPase dMiro is required for axonal transport of mitochondria to *Drosophila* synapses. Neuron.

[81] Wang X, Schwarz TL (2009). The mechanism of Ca2+ -dependent regulation of kinesin-mediated mitochondrial motility. Cell.

[82] Saxton WM, Hollenbeck PJ (2012). The axonal transport of mitochondria. J. Cell Sci..

[83] Lee CA, Chin LS, Li L (2018). Hypertonia-linked protein Trak1 functions with mitofusins to promote mitochondrial tethering and fusion. Protein Cell.

[84] Misko A, Jiang S, Wegorzewska I, Milbrandt J, Baloh RH (2010). Mitofusin 2 is necessary for transport of axonal mitochondria and interacts with the Miro/Milton complex. J. Neurosci..

[85] Cartoni R (2016). The mammalian-specific protein Armcx1 regulates mitochondrial transport during axon regeneration. Neuron.

[86] Ringer K (2018). The large GTPase Mx1 binds Kif5B for cargo transport along microtubules. Traffic.

[87] Nath S (2007). Kif5B and Kifc1 interact and are required for motility and fission of early endocytic vesicles in mouse liver. Mol. Biol. Cell.

[88] Delevoye C (2014). Recycling endosome tubule morphogenesis from sorting endosomes requires the kinesin motor KIF13A. Cell Rep..

[89] Rappsilber J, Mann M, Ishihama Y (2007). Protocol for micro-purification, enrichment, pre-fractionation and storage of peptides for proteomics using StageTips. Nat. Protoc..

[90] Cox J, Mann M (2008). MaxQuant enables high peptide identification rates, individualized p.p.b.-range mass accuracies and proteome-wide protein quantification. Nat. Biotechnol..

[91] Cox J (2011). Andromeda: a peptide search engine integrated into the MaxQuant environment. J. Proteome Res..

[92] McCall MN, Bolstad BM, Irizarry RA (2010). Frozen robust multiarray analysis (fRMA). Biostatistics.

[93] Smoot ME, Ono K, Ruscheinski J, Wang PL, Ideker T (2010). Cytoscape 2.8: new features for data integration and network visualization. Bioinformatics.

[94] Subramanian A (2005). Gene set enrichment analysis: a knowledge-based approach for interpreting genome-wide expression profiles. Proc. Natl Acad. Sci. USA.

[95] Covill-Cooke C (2020). Peroxisomal fission is modulated by the mitochondrial Rho-GTPases, Miro1 and Miro2. EMBO Rep..

[96] Manders EMM, Verbeek FJ, Aten JA (1993). Measurement of co-localization of objects in dual-colour confocal images. J. Microsc.

[97] Yang TT (2018). Super-resolution architecture of mammalian centriole distal appendages reveals distinct blade and matrix functional components. Nat. Commun..

[98] Chong WM (2020). Super-resolution microscopy reveals coupling between mammalian centriole subdistal appendages and distal appendages. Elife.

[99] Pagliuso A (2016). A role for septin 2 in Drp1‐mediated mitochondrial fission. EMBO Rep..

[100] De Vos KJ, Sheetz MP (2007). Visualization and quantification of mitochondrial dynamics in living animal cells. Methods Cell Biol..

[101] Mitra K, Lippincott‐Schwartz J (2010). Analysis of mitochondrial dynamics and functions using imaging approaches. Curr. Protoc. Cell Biol..

[102] Vizcaíno JA (2016). 2016 update of the PRIDE database and its related tools. Nucleic Acids Res..

