# Supplementary material for: Spatial and temporal dynamics of ATP synthase from mitochondria toward the cell surface
Source: Commun Biol. 2023 Apr 18;6:427. doi: 10.1038/s42003-023-04785-3 (PMC10113393; doi:10.1038/s42003-023-04785-3)
Supplement: Supplementary file 1 — Supplementary Information [file 42003_2023_4785_MOESM1_ESM.pdf]

Supplementary Information

Spatial and temporal dynamics of ATP synthase from mitochondria toward the cell surface

Yi-Wen Chang[1], T. Tony Yang[2,3], Min-Chun Chen[1], Y-geh Liaw[1], Chieh-Fan Yin[1], Xiu-Qi Lin-Yan[1], Ting-Yu Huang[1], Jen-Tzu Hou[1], Yi-Hsuan Hung[1], Chia-Lang Hsu[1,4], Hsuan-Cheng Huang[5,*], Hsueh-Fen Juan[1,3,6,*]

[1]Department of Life Science, Institute of Molecular and Cellular Biology, National Taiwan University, Taipei, 106, Taiwan
[2]Department of Electrical Engineering, National Taiwan University, Taipei, 106, Taiwan
[3]Graduate Institute of Biomedical Electronics and Bioinformatics, National Taiwan University, Taipei, 106, Taiwan
[4]Department of Medical Research, National Taiwan University Hospital, Taipei 100, Taiwan
[5]Institute of Biomedical Informatics, National Yang Ming Chiao Tung University, Taipei, 112, Taiwan
[6]Center for Computational and Systems Biology, National Taiwan University, Taipei, 106, Taiwan

*Correspondence should be addressed to H.-C.H (hsuancheng@nycu.edu.tw) or H.-F.J. (yukijuan@ntu.edu.tw).

Correspondence:
Hsuan-Cheng Huang, Ph.D.
Institute of Biomedical Informatics, National Yang Ming Chiao Tung University, No.155, Sec.2, Linong Street, Taipei 112, Taiwan; Tel: +886-2-28267357; Fax: +886-2-28202508; E-mail: hsuancheng@nycu.edu.tw

Hsueh-Fen Juan, Ph.D.
Department of Life Science, Graduate Institute of Biomedical Electronics and Bioinformatics, Center for Computational and Systems Biology, National Taiwan University, No. 1, Sec. 4, Roosevelt Road, Taipei 106, Taiwan; Tel: +886-2-33664536; Fax: +886-2-23673374; E-mail: yukijuan@ntu.edu.tw

This PDF file includes **Supplementary Figs. 1-10**.

Supplementary Fig. 1

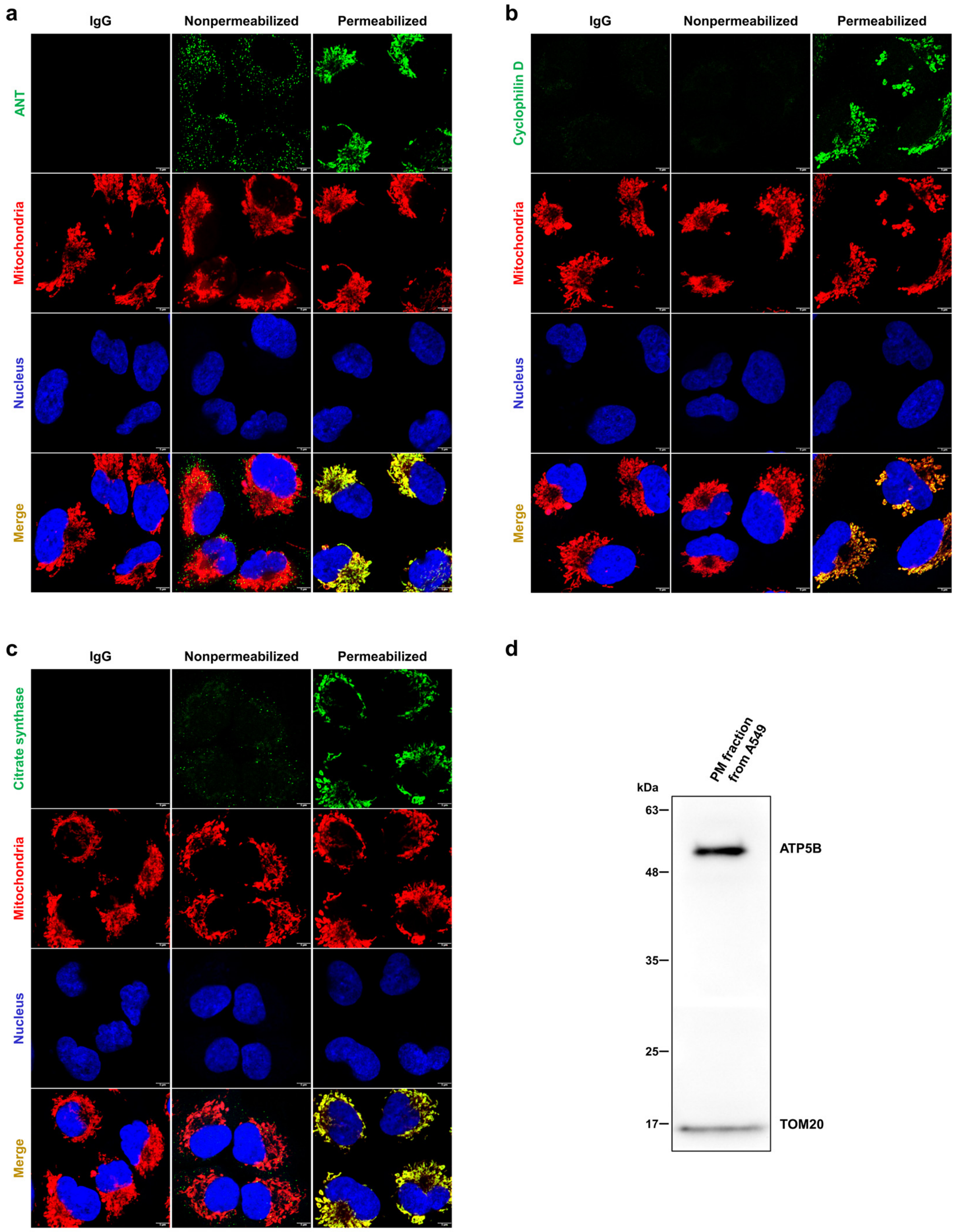

Supplementary Fig. 1: Not only ATP synthase but also other mitochondrial proteins ectopically express on the cell surface of cancer cells. a-c The abundance of ANT (**a**), Cyclophilin D (**b**), or Citrate synthase (**c**) on the cell surface of non-permeabilized cells was determined using confocal microscopy (100×). Cells permeabilized by 0.1% triton X-100 were used to observe the distribution pattern of these proteins in the mitochondria. IgG served as the negative control. Scale bars, 5 μm. **d** The expression levels of ATP5B and TOM20 in the plasma membrane fraction of A549 cells were detected by western blotting.

Supplementary Fig. 2

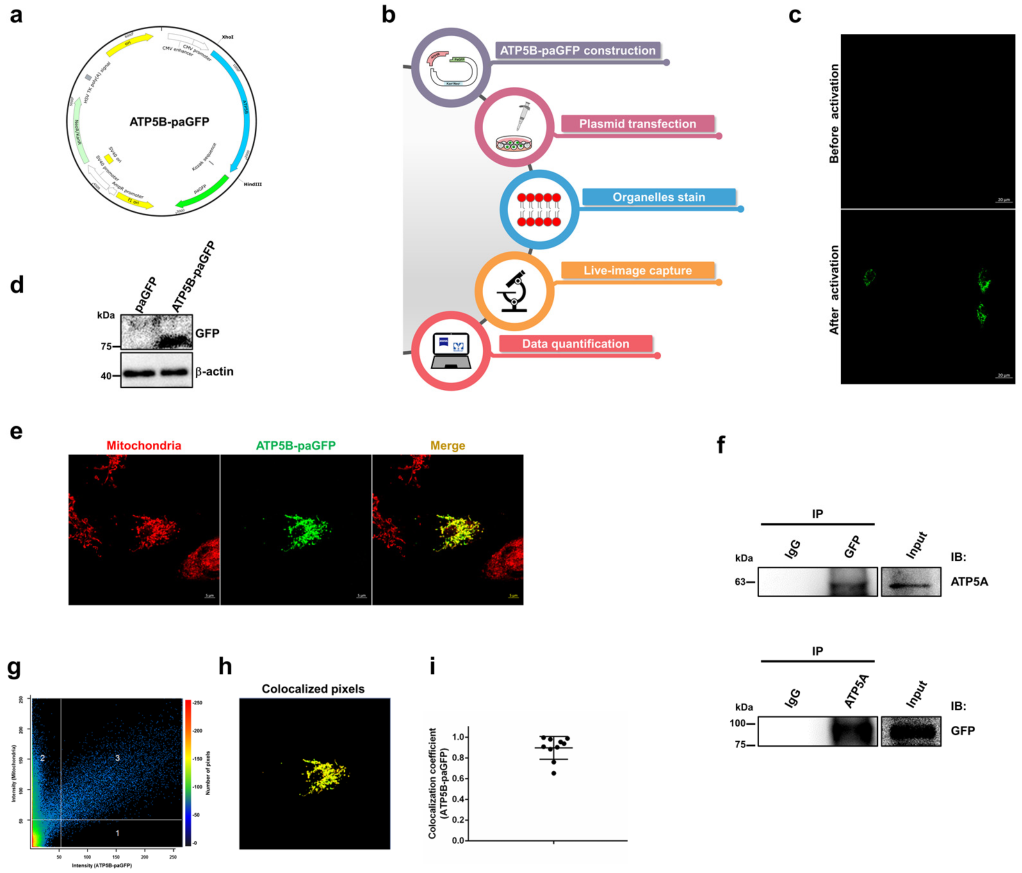

Supplementary Fig. 2: The spatial localization of ATP synthase in live A549 cells is determined using the ATP5B-paGFP fusion protein. a The sequence map for ATP5B-paGFP recombinant construct. **b** Schematic representation of the real-time tracing system of ATP synthase. **c** The ATP synthase β-paGFP signal in live cells was photoactivated with a 405 nm laser using confocal microscopy. Scale bars, 20 μm. **d** The expression levels of ATP synthase β-paGFP fusion proteins in transfected cells were detected by western blotting using antibody against GFP. **e** The subcellular localizations of intracellular ATP synthase β-paGFP fusion protein (green) and the mitochondria (MitoTracker; red) in live cells were detected using confocal microscopy. The colocalization of these two signals is shown as yellow signals in the merged images. Scale bars, 5 μm. **f** Co-immunoprecipitation with anti-GFP antibody or anti-ATP5A, followed by western blotting using the indicated antibodies, was performed to verify whether ATP synthase β subunit-paGFP fusion protein assembled in the F1 catalytic core of the enzyme. **g** The fluorescent signals of ATP synthase β-paGFP fusion protein (green; Q1), mitochondria (red; Q2), and colocalization (yellow; Q3) were determined by ZEN software and are shown as the scatter plot. **h** The pixels in Q3 were false colored to yellow to easily visualized the colocalized pixels in the fluorescence image. **i** The colocalization coefficient of ATP5B-

paGFP (colocalized pixels versus total ATP5B-paGFP pixels in one cell) was presented as mean ± SD in bar chart (n = 10). * $p < 0.05$, ** $p < 0.01$, *** $p < 0.001$.

Supplementary Fig. 3

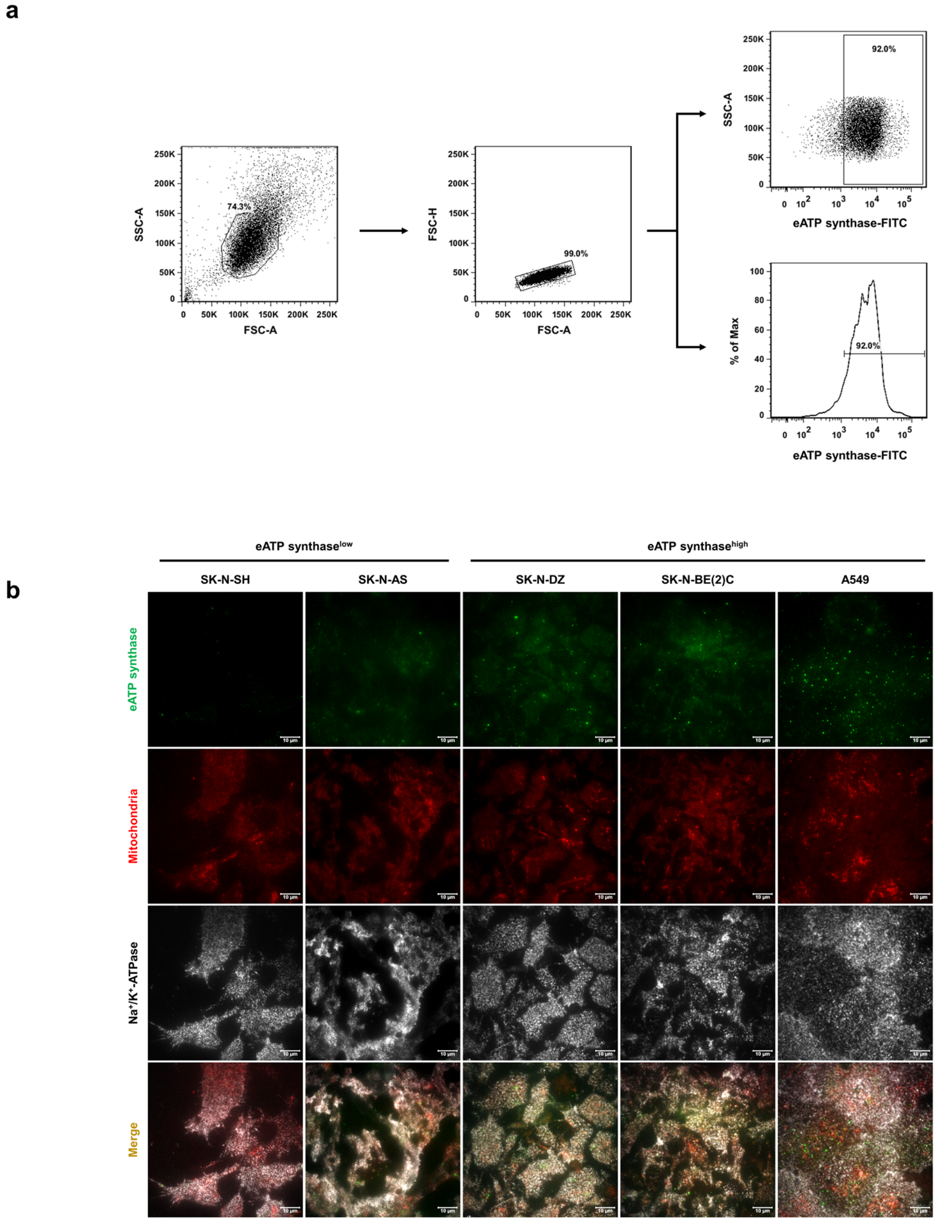

Supplementary Fig. 3: The presence of ATP synthase on the plasma membrane of various cancer cells. a Gating strategy for detecting the abundance of eATP synthase in cancer cells in this study. **b** The signals of eATP synthase on the basal plasma membrane of different cancer cell lines were labeled by the anti-ATP synthase antibody, followed by hybridization with Alexa 488 anti-mouse IgG (green), and observed using TIRF microscopy. MitoTracker (red) labeled mitochondria and anti-Na^+/K^+-ATPase antibody followed by hybridization with Alexa 647 anti-Rabbit IgG (white) labeled the plasma membrane. The overlay showed the colocalization of these three signals. Scale bars, 10 μm.

Supplementary Fig. 4

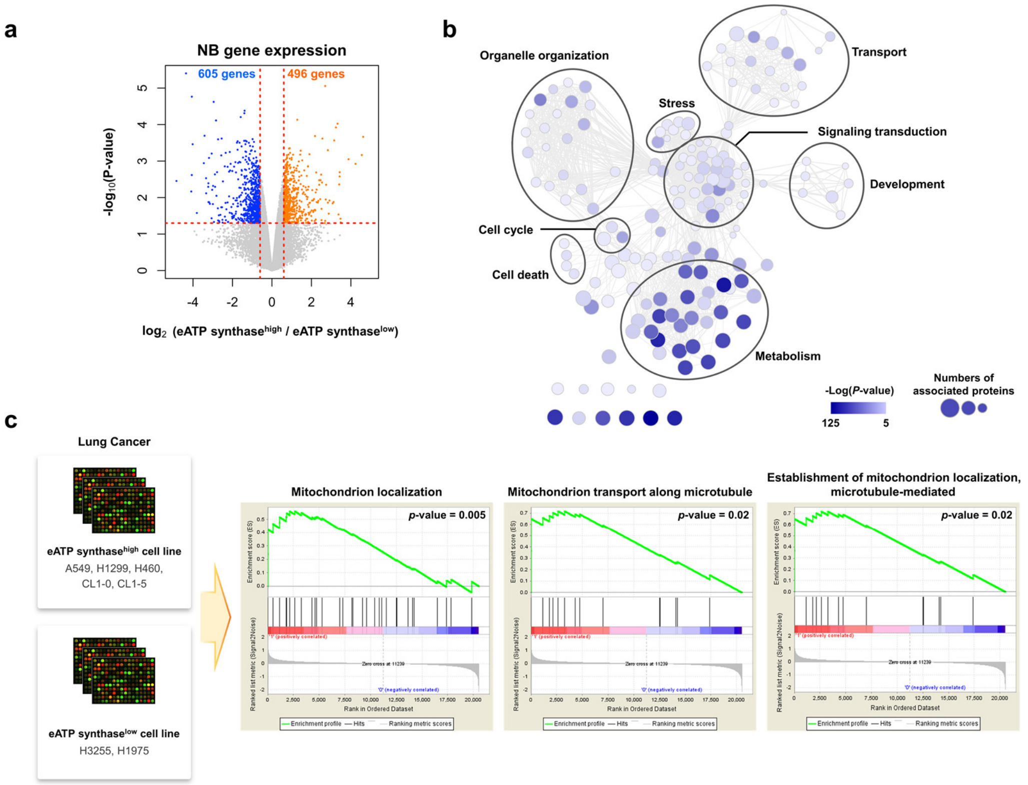

Supplementary Fig. 4: The potential mechanism of ATP synthase trafficking to the cell surface. a Volcano plot depict the gene expression differences between eATP synthase^{high} and eATP synthase^{low} cell lines. The orange points represent the significantly up-regulated genes and the blue points represent the significantly down-regulated genes ($p < 0.05$ and fold-change > 1.5). **b** Functional enrichment network of the eATP synthase-related genes. Nodes represent gene ontology terms (GO terms) which were statistically enriched in the genes with differential expression. Links connecting nodes indicate the relatedness between nodes. **c** Gene set enrichment analysis (GSEA) was conducted on the gene expression profiles of the eATP synthase^{high} and eATP synthase^{low} lung cancer cell lines. The gene sets associated with mitochondrial trafficking were significantly positively enriched (*p*-values < 0.05). Enrichment scores are 0.72, 0.72, 0.56 for GO terms “Mitochondrion transport along microtubule”, “Establishment of mitochondrion localization, microtubule-mediated” and “Mitochondrion localization”.

Supplementary Fig. 5

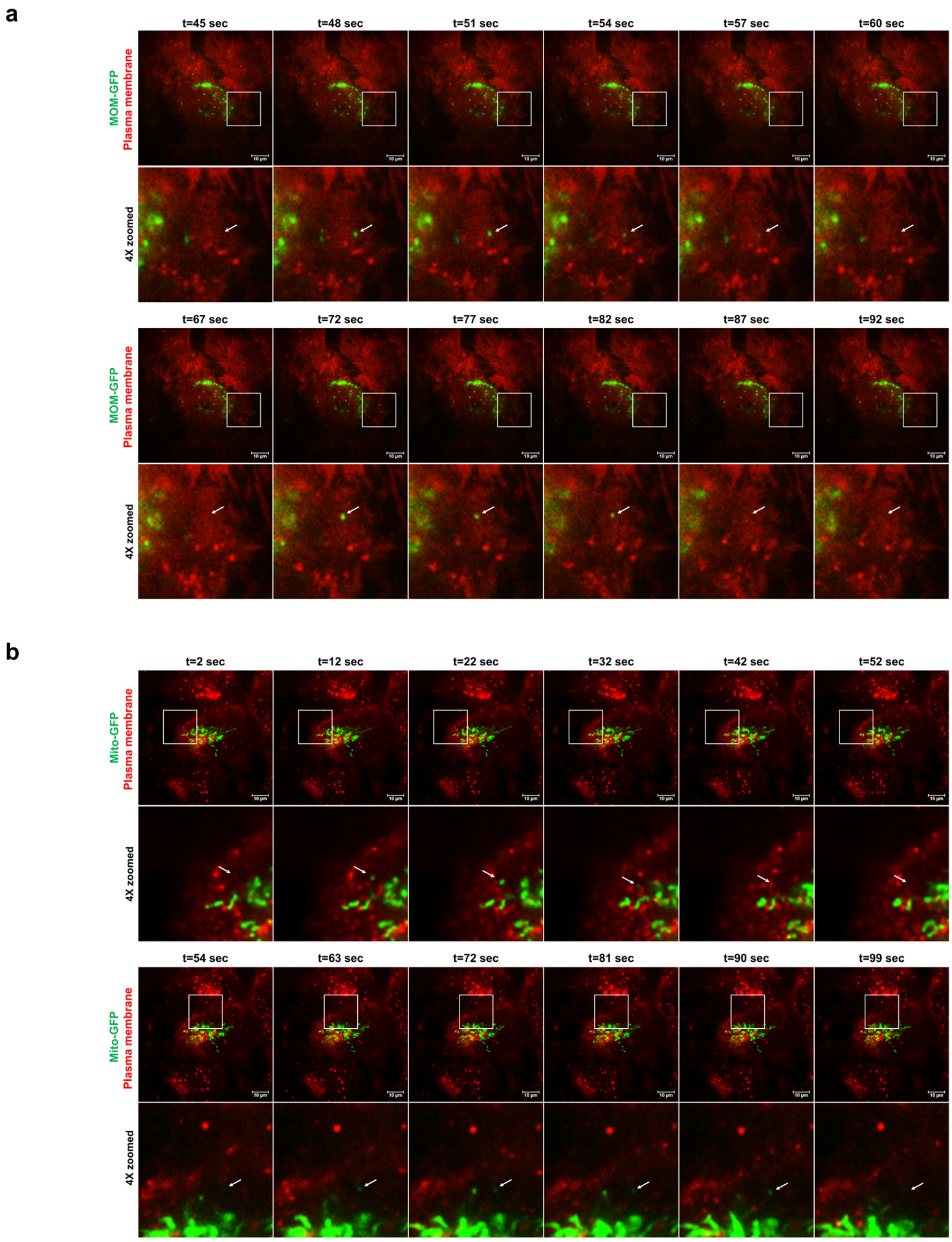

Supplementary Fig. 5: Real-time mitochondria-PM fusion events were observed at the basal surface of cells using TIRF microscopy. a, b Time-lapse images of GFP-labeled mitochondrial outer membrane (MOM-GFP; a) and mitochondrial inner membrane (Mito-GFP; b) in live A549 cells were visualized using TIRF microscopy. The plasma membrane was labeled using CellMask (1:10,000 dilution; red). White arrows on the enlarged images indicating the spread out of GFP signals represent the possible fusion events. The capture times in the recording period are shown. Scale bars, 10 μm.

Supplementary Fig. 6

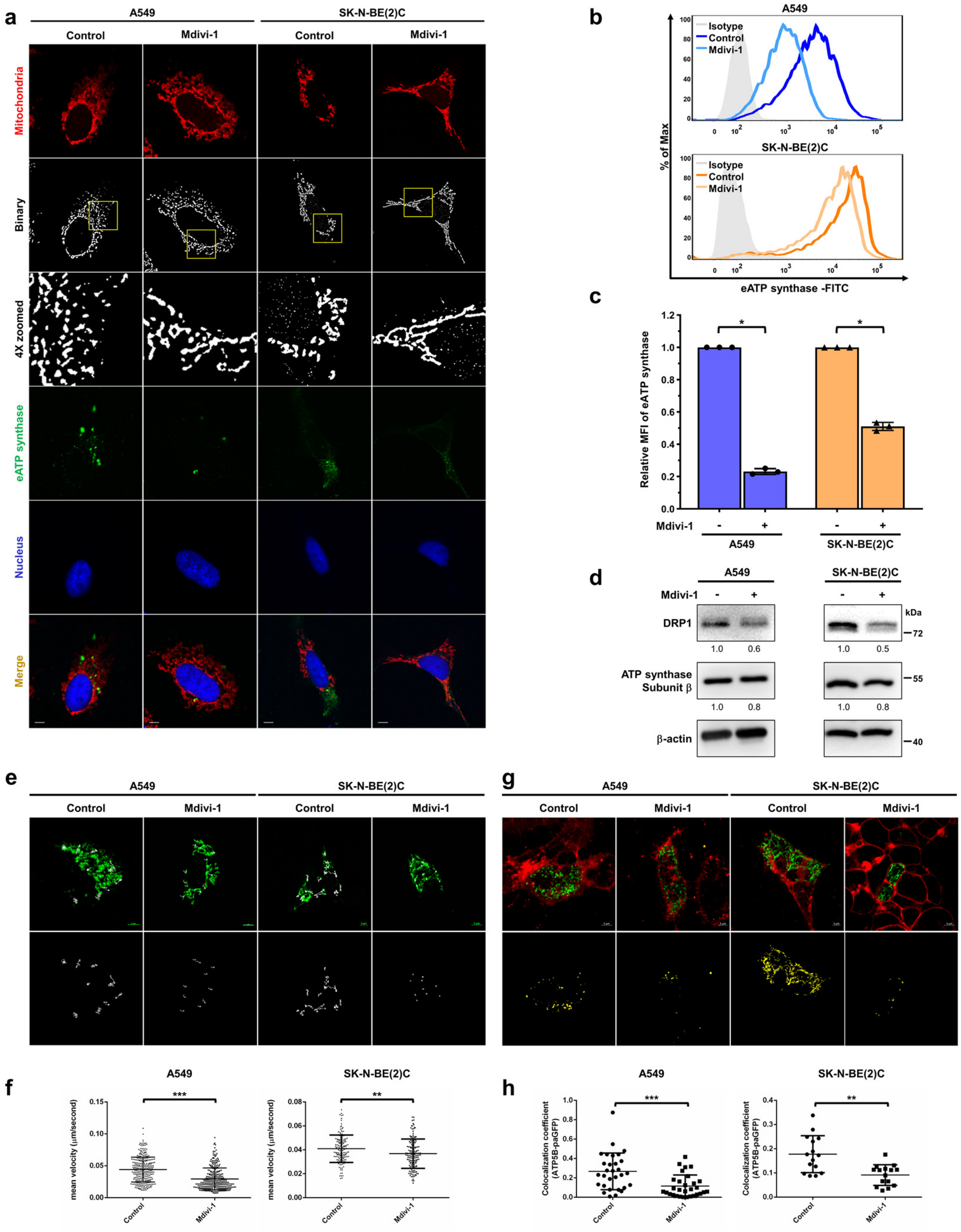

Supplementary Fig. 6: Inhibition of mitochondrial fission suppresses the transport of ATP synthase toward the cell surface. A549 and SK-N-BE(2)C cells were treated with DMSO or Mdivi-1 (30 μM) in (a-h). **a** Mitochondrial morphology and the expression of eATP synthase on the cell surface of A549 and SK-N-BE(2)C cells were visualized using confocal microscopy (100×) and Mitotracker (red) or the antibody against the ATP synthase complex, followed by hybridization with Alexa 488 anti-mouse IgG (green). The binary images shown were processed using Icy software. Scale bars, 5 μm. **b** The abundance of eATP synthase was determined via flow cytometry using anti-ATP synthase antibody in nonpermeable cells. **c** The relative MFI of eATP synthase based on the flow cytometry data is displayed as a bar chart. Data presented are the mean ± SD (n = 3). **d** The protein levels of Drp1 and ATP synthase subunit β were verified using western blotting in the DMSO and Mdivi-1-treated groups. The intensity of the bands was normalized to that of β-actin and relative to the expression of each protein in the DMSO-treated group. **e** The movement of the ATP synthase subunit β-paGFP fusion protein in the DMSO or Mdivi-1 group was recorded for 15 min via confocal microscopy (upper). Their tracks are shown as white lines and were analyzed using Metamorph software (lower). **f** The dot plot represents the quantification of the translocation velocity of both A549 and SK-N-BE(2)C cells (n = 30). **g** The localization of ATP synthase subunit β-paGFP fusion protein (green) and PM (CellMask; red) were determined using confocal microscopy. The colocalization of these two signals is shown as yellow fluorescence in the merged images (upper), and was further processed using ZEN software (lower). **h** The dot plot shows the colocalization coefficient (colocalization fluorescent area versus total ATP synthase subunit β-paGFP fluorescent area) (n = 30). Values shown are the mean ± SD. * $p < 0.05$, ** $p < 0.01$, *** $p < 0.001$

Supplementary Fig. 7

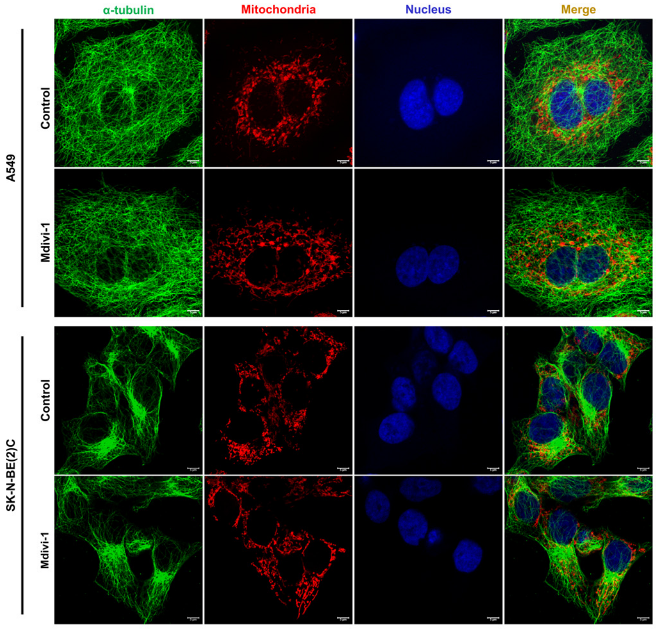

Supplementary Fig. 7: Inhibition of mitochondrial did not prominently affect microtubule polymerization. Anti-α-tubulin antibody, followed by hybridization with Alexa 488 anti-mouse IgG (green), was used to determine the microtubule polymerization in the cells treated with or without Mdivi-1. Mitochondria morphology was confirmed using Mitotracker (red) staining. Scale bars, 5 μm.

Supplementary Fig. 8

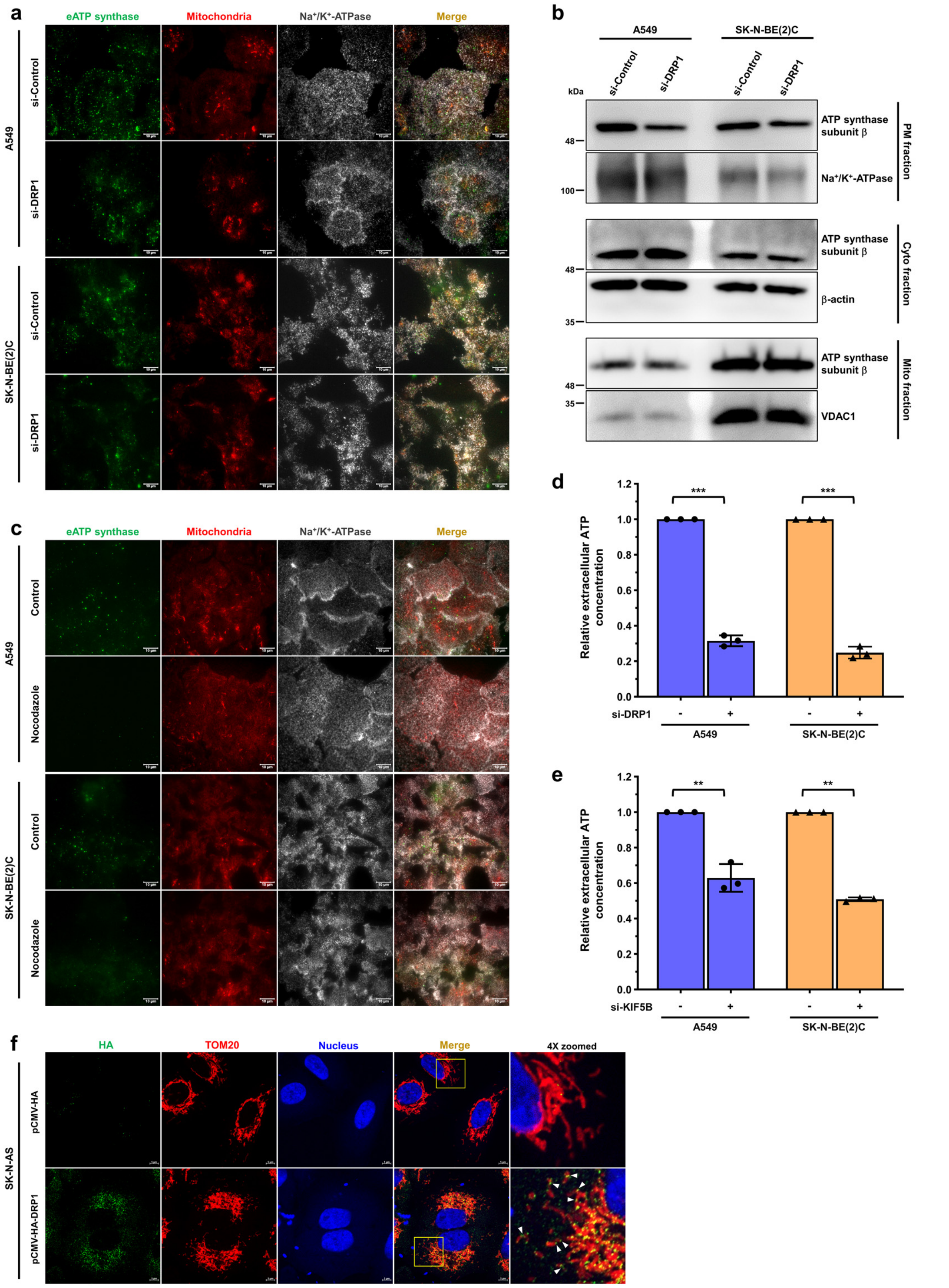

Supplementary Fig. 8: DRP1-mediated ATP synthase transportation along microtubules regulates the abundance of eATP synthase on the cell surface and extracellular ATP production. a, c The signals of eATP synthase on the basal plasma membrane siDRP1-silencing (**a**) or nocodazole-treated (**c**) cells were determined by TIRF microscopy. Non-permeabilized cells were labeled by the anti-ATP synthase antibody, followed by hybridization

with Alexa 488 anti-mouse IgG (green), and observed using TIRF microscopy. MitoTracker (red) labeled mitochondria and anti-Na^+/K^+-ATPase antibody followed by hybridization with Alexa 647 anti-Rabbit IgG (white) labeled the plasma membrane. The overlay showed the colocalization of these three signals. Scale bars, 10 μm. **b** The expression levels of ATP synthase in the plasma membrane (PM), cytosolic (Cyto), and mitochondrial (Mito) fractions were confirmed using western blotting. **d, e** The extracellular ATP concentration in the conditioned medium of DRP1 (**d**) or KIF5B-knockdown cells (**e**) was determined by ATP bioluminescence assay, respectively. The relative concentration was normalized according to the number of cells and the amount of extracellular ATP of si-Control cells. **f** The overexpression efficiency of HA-DRP1-transfected SK-N-AS cells was confirmed by immunofluorescence. Anti-HA antibody, followed by hybridization with Alexa 488-conjugated secondary antibody (green), was used to determine the expression and the distribution of HA-DRP1, and anti-TOM20 antibody followed by hybridization with Alexa 555-conjugated secondary antibody (red) labeled mitochondria. Scale bars, 5 μm.

Supplementary Fig. 9

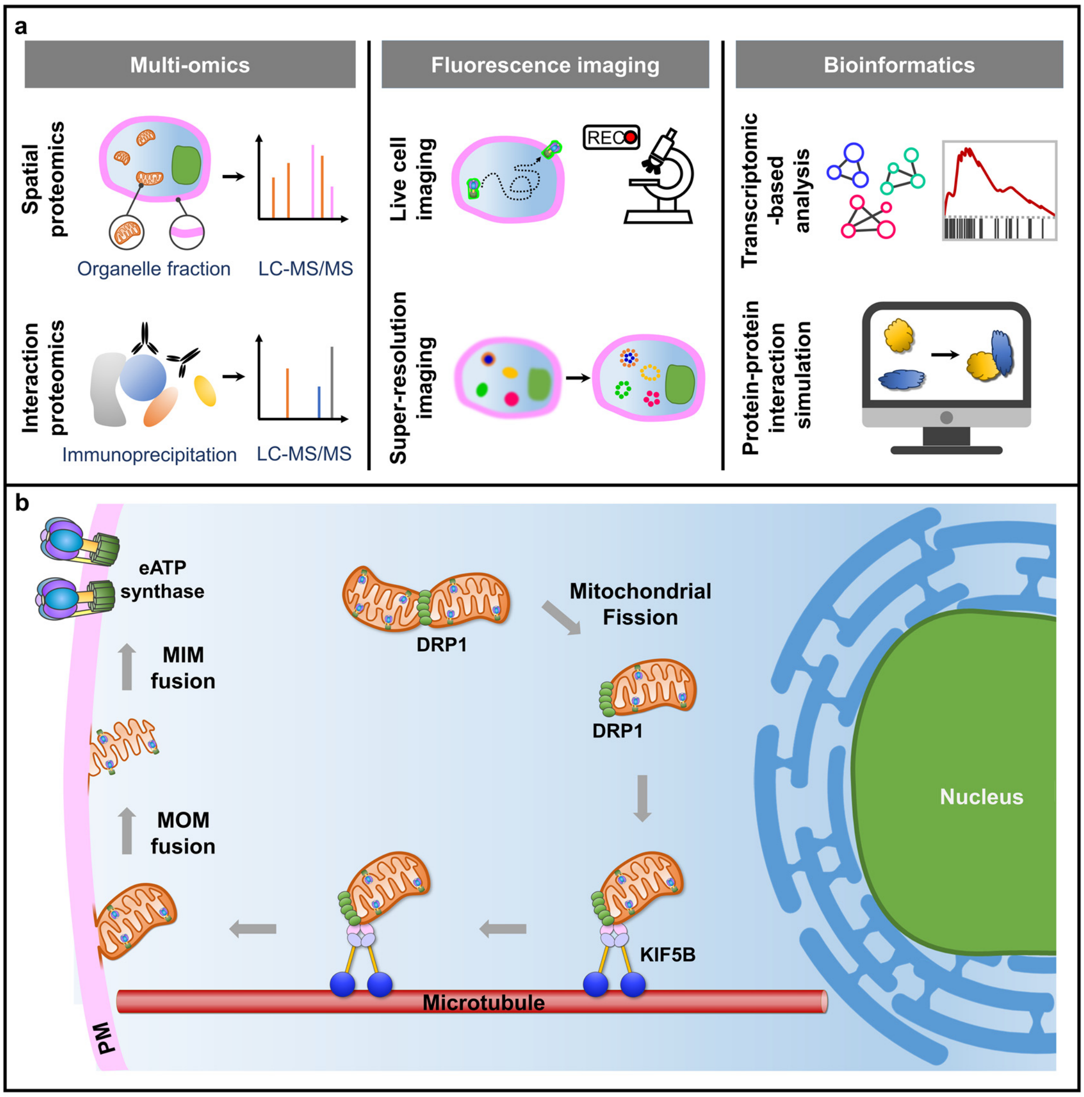

Supplementary Fig. 9: Overall schema of this study. a Schematic of experiment design for identifying the trafficking mechanism of mitochondrial ATP synthase. **b** Model of ATP synthase trafficking pathway from mitochondria to cell surface**.** Ectopic ATP synthase complex is assembled in mitochondria and transported along microtubules to the cell surface dependent on KIF5B and DRP1. The mitochondrial outer membrane and inner membrane attach to the plasma membrane in turn to anchor ATP syntheses on the cell surface.

Supplementary Fig. 10

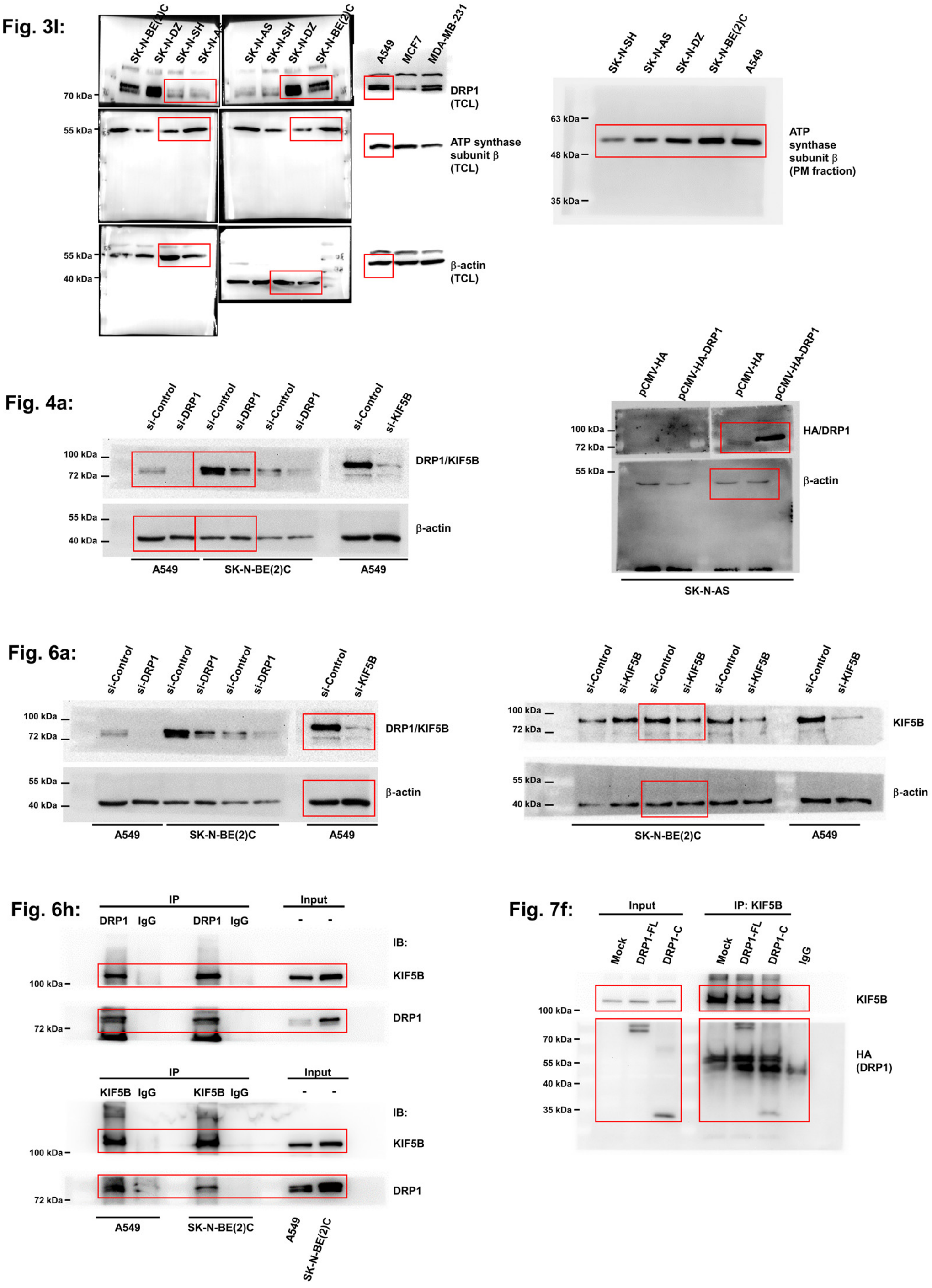

Supplementary Fig. 10 (continued)

Fig. S1d:

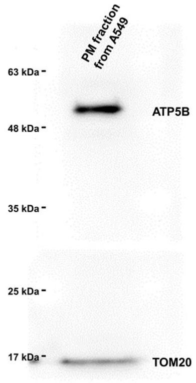

Fig. S2f:

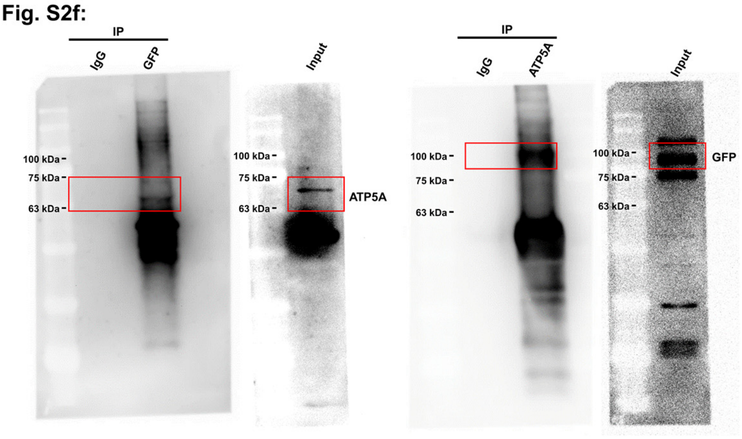

Fig. S6d:

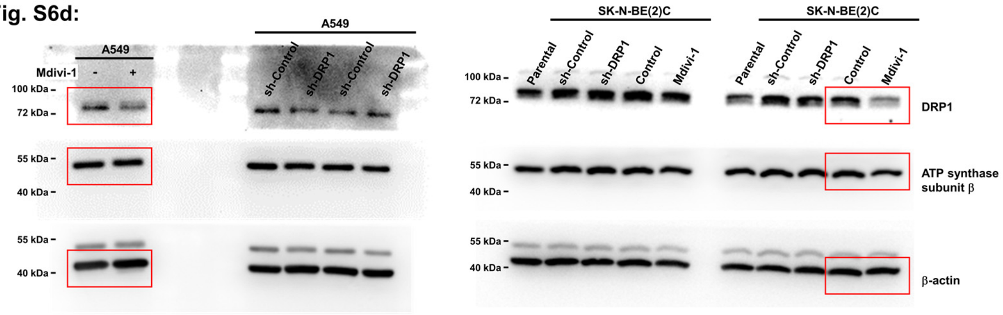

Fig. S8b:

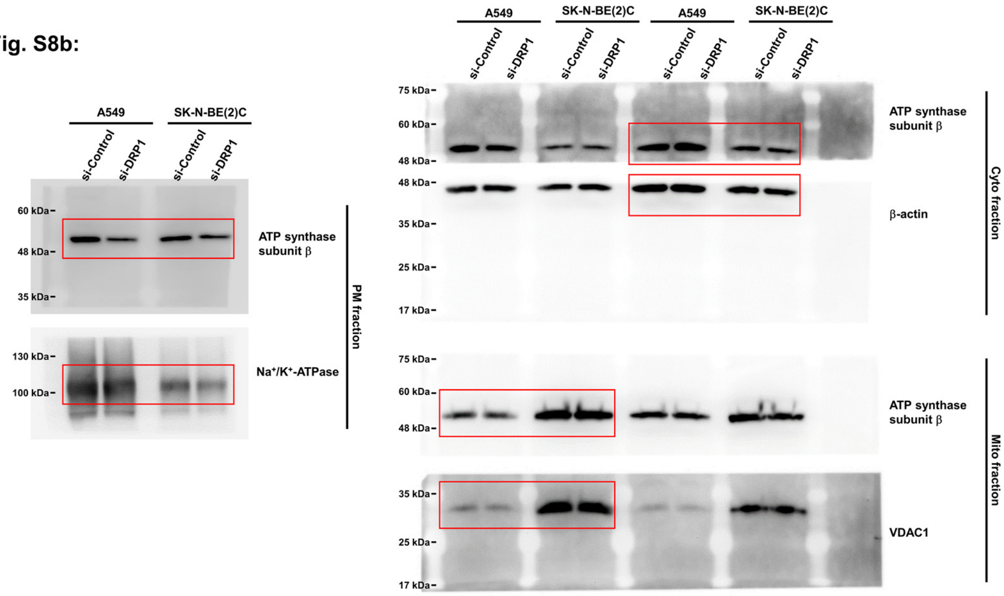

Supplementary Fig. 10: Uncropped and unedited blot/gel images.
