# Supplementary material for: Spatial and temporal dynamics of ATP synthase from mitochondria toward the cell surface
Source: Commun Biol. 2023 Apr 18;6:427. doi: 10.1038/s42003-023-04785-3 (PMC10113393; doi:10.1038/s42003-023-04785-3)
Supplement: Supplementary file 2 — Description of Additional Supplementary Files [file 42003_2023_4785_MOESM2_ESM.pdf]

**Description of Additional Supplementary Files**

**File name:** Supplementary Data 1
**Description:** Mitochondrial proteome profile of A549 lung cancer cells

**File name:** Supplementary Data 2
**Description:** Plasma membrane proteome profile of A549 lung cancer cells

**File name:** Supplementary Data 3
**Description:** The list of proteins located on both mitochondria and plasma membrane in A549 lung cancer cells

**File name:** Supplementary Data 4
**Description:** The list of the significantly differential genes in eATP synthase$^{high}$ compared to eATP synthase$^{low}$-expressing cell

**File name:** Supplementary Data 5
**Description:** Table for Gene Ontology analysis of differential expressed genes in eATP$^{high}$ compared to eATP$^{low}$-expressing cells

**File name:** Supplementary Data 6
**Description:** The list of proteins of KIF5B-interactomic profiling in A549 lung cancer cells

**File name:** Supplementary Data 7
**Description:** Table for subcellular location of KIF5B-interactomic proteins in A549 lung cancer cells

**File name:** Supplementary Data 8
**Description:** Source data used for plotting the graphs and charts presented in the manuscript.

**File name:** Supplementary Movie 1
**Description:** Real-time tracing of the subcellular trafficking of the ATP5B-paGFP fusion protein (field 1). See also Fig. 1e; left

**File name:** Supplementary Movie 2
**Description:** Real-time monitoring of the subcellular trafficking of the ATP5B-paGFP fusion protein (field 2). See also Fig. 1e; right

**File name:** Supplementary Movie 3
**Description:** Real-time tracing of the subcellular trafficking of the ATP5B-paGFP fusion protein (field 3). See also Fig. 1f

**File name:** Supplementary Movie 4
**Description:** Real-time tracing of the subcellular trafficking of the ATP5B-paGFP fusion protein.

**File name:** Supplementary Movie 5
**Description:** Real-time tracing of the subcellular trafficking of the ATP5B-paGFP fusion protein and mitochondria. See also Fig. 2f

**File name:** Supplementary Movie 6
**Description:** Real-time tracing of the subcellular trafficking of the ATP5B-paGFP fusion protein and mitochondria.

**File name:** Supplementary Movie 7
**Description:** Real-time monitoring mitochondrial outer membrane-plasma membrane fusion event.

**File name:** Supplementary Movie 8
**Description:** Real-time monitoring mitochondrial inner membrane-plasma membrane fusion event.

**File name:** Supplementary Movie 9
**Description:** Observation of mitochondrial outer membrane-plasma membrane fusion event in live cells using TIRF microscopy.

**File name:** Supplementary Movie 10

**Description:** Observation of mitochondrial inner membrane-plasma membrane fusion event in live cells using TIRF microscopy.
