# Supplementary material for: Spatial and temporal dynamics of ATP synthase from mitochondria toward the cell surface
Source: Commun Biol. 2023 Apr 18;6:427. doi: 10.1038/s42003-023-04785-3 (PMC10113393; doi:10.1038/s42003-023-04785-3)
Supplement: Supplementary file 21 — Reporting Summary [file 42003_2023_4785_MOESM21_ESM.pdf]

nature portfolio

Corresponding author(s): Hsueh-Fen Juan

Last updated by author(s): Mar 20, 2023

Reporting Summary

Nature Portfolio wishes to improve the reproducibility of the work that we publish. This form provides structure for consistency and transparency in reporting. For further information on Nature Portfolio policies, see our Editorial Policies and the Editorial Policy Checklist.

Statistics

For all statistical analyses, confirm that the following items are present in the figure legend, table legend, main text, or Methods section.

n/a	Confirmed	
☐	☒	The exact sample size (*n*) for each experimental group/condition, given as a discrete number and unit of measurement
☐	☒	A statement on whether measurements were taken from distinct samples or whether the same sample was measured repeatedly
☐	☒	The statistical test(s) used AND whether they are one- or two-sided<br>*Only common tests should be described solely by name; describe more complex techniques in the Methods section.*
☐	☒	A description of all covariates tested
☐	☒	A description of any assumptions or corrections, such as tests of normality and adjustment for multiple comparisons
☐	☒	A full description of the statistical parameters including central tendency (e.g. means) or other basic estimates (e.g. regression coefficient) AND variation (e.g. standard deviation) or associated estimates of uncertainty (e.g. confidence intervals)
☐	☒	For null hypothesis testing, the test statistic (e.g. *F*, *t*, *r*) with confidence intervals, effect sizes, degrees of freedom and *P* value noted<br>*Give P values as exact values whenever suitable.*
☒	☐	For Bayesian analysis, information on the choice of priors and Markov chain Monte Carlo settings
☒	☐	For hierarchical and complex designs, identification of the appropriate level for tests and full reporting of outcomes
☒	☐	Estimates of effect sizes (e.g. Cohen's *d*, Pearson's *r*), indicating how they were calculated

Our web collection on statistics for biologists contains articles on many of the points above.

Software and code

Policy information about availability of computer code

Data collection	1. Mass spectrometry data were obtained from LTQ-Orbitrap XL (Thermo Electron) equipped with a nanoACQUITY ultra-performance liquid chromatography system (Waters).<br>2. The gene expression profiles used in this study were mined from Gene Expression Omnibus (GEO) (RRID:SCR_005012) using the R package “GEOquery” with accession number GSE78061.<br>3. The protein bands were analyzed using FluorChem M (ProteinSimple).<br>4. Immunofluorescent images were captured using a Zeiss LSM780 confocal microscope (Zeiss).<br>5. The super-resolution images were performed on the direct stochastic optical reconstruction microscopy (dSTORM) system.<br>6. Flow cytometry data were collected using a BD FACSCanto II instrument (BD Biosciences).
Data analysis	1. The raw MS spectra data were analyzed using MaxQuant (RRID:SCR_014485) for peak detection and protein identification (version 1.3.0.5. for PM proteins; version 1.5.2.8 for mitochondrial proteins and co-immunoprecipitation).<br>2. The differentially expressed genes were identified using R package: limma.<br>3. The signal of ectopic ATP synthase were analyzed using ImageJ (ImageJ)<br>4. Mitochondrial perimeter and area were quantified using Icy (deChaumont et al, 2012)<br>5. The movement of fluorescent signals over time were measured using Metamorph software (Molecular Devices).<br>6. The colocalization coefficient was determined using Zen 2010 software (Zeiss)<br>7. Flow cytometry data were analyzed using BD FACSCanto II Software(BD Biosciences) and FlowJo 7.6.1 (BD Biosciences)<br>8. All plots in this manuscript were generated by GraphPad Prism 9 (GraphPad Software Inc) or Excel (2016).

For manuscripts utilizing custom algorithms or software that are central to the research but not yet described in published literature, software must be made available to editors and reviewers. We strongly encourage code deposition in a community repository (e.g. GitHub). See the Nature Portfolio guidelines for submitting code & software for further information.

Data

Policy information about availability of data

All manuscripts must include a data availability statement. This statement should provide the following information, where applicable:

- Accession codes, unique identifiers, or web links for publicly available datasets
- A description of any restrictions on data availability
- For clinical datasets or third party data, please ensure that the statement adheres to our policy

All original mass spectrometry data have been deposited to the ProteomeXchange Consortium via the PRIDE partner repository with the dataset identifiers PXD006791 15 (plasma membrane proteome), and PXD007036 (mitochondrial proteome).

Human research participants

Policy information about studies involving human research participants and Sex and Gender in Research.

Reporting on sex and gender	NA
Population characteristics	NA
Recruitment	NA
Ethics oversight	NA

Note that full information on the approval of the study protocol must also be provided in the manuscript.

Field-specific reporting

Please select the one below that is the best fit for your research. If you are not sure, read the appropriate sections before making your selection.

☒ Life sciences ☐ Behavioural & social sciences ☐ Ecological, evolutionary & environmental sciences

For a reference copy of the document with all sections, see nature.com/documents/nr-reporting-summary-flat.pdf

Life sciences study design

All studies must disclose on these points even when the disclosure is negative.

Sample size	No sample-size calculation was performed.
Data exclusions	No data were excluded from the analyses.
Replication	In all experiments, three technical replicates were performed for each of the two independent biological replicates. All findings at replication were successful.
Randomization	Cells were parallel seeded and randomly assigned to different treatments.
Blinding	Blinding was not applied during experiments.

Reporting for specific materials, systems and methods

We require information from authors about some types of materials, experimental systems and methods used in many studies. Here, indicate whether each material, system or method listed is relevant to your study. If you are not sure if a list item applies to your research, read the appropriate section before selecting a response.

Materials & experimental systems

n/a	Involved in the study
	☒ Antibodies
	☒ Eukaryotic cell lines
☒	☐ Palaeontology and archaeology
☒	☐ Animals and other organisms
☒	☐ Clinical data
☒	☐ Dual use research of concern

Methods

n/a	Involved in the study
☒	☐ ChIP-seq
	☒ Flow cytometry
☒	☐ MRI-based neuroimaging

Antibodies

Antibodies used

1. The primary antibodies against the following proteins were used in this study: ATP synthase complex (Immunofluore.cence, Flow cytometry; Abcam ab109867), KIF5B (Western blotting, Immunoprecipitation; Abcam ab167429), DRP1 (Western blotting, Immunoprecipitation; Abcam ab56788) , Sodium Potassium ATPase (Immunofluorescence; Abcam ab 76020), TOMM20 (Immunofluorescence, Western blotting; Abcam ab186735), alpha-tubulin (Immunofluorescence; GeneTex GTX628802), ATP synthase beta subunit (Western blotting; GeneTex GTX84845), HA-tag (Western blotting, Immunoprecipitation; BioLegend 901501), E-cadherin (Immunofluorescence; Merck Millipore MAB3199Z), and Actin (Western blotting; Merck Millipore MAB1501).
2. The secondary antibodies used in this study are listed as follows: Goat Anti-Mouse IgG H&L (HRP) (Western blotting; Abcam ab97023), Goat Anti-Rabbit IgG H&L (HRP) (Western blotting; Abcam ab97051), Alexa488-conjugated Goat anti-mouse IgG (Immunofluorescence; Invitrogen Cat#A11001), Alexa488-conjugated Goat anti-rabbit IgG (Immunofluorescence; Invitrogen Cat#A11008), Alexa647-conjugated Goat anti-mouse IgG (Immunofluorescence; Invitrogen A21236), Alexa647-conjugated Goat anti-rabbit IgG (Immunofluorescence; Immunofluorescence; Invitrogen A21245), Cy3B Maleimide (Immunofluorescence; GE Healthcare PA63131).

Validation

1. Anti-ATP synthase Immunocapture antibody [12F4AD8AF8] (Abcam ab109867) antibody reacts with human species and is suitable for Flow Cyt. This antibody was validated using flow cytometry and immunofluorescence in this study.
2. Recombinant Anti-KIF5B antibody [EPR10276(B)] antibody (Abcam ab167429) reacts with mouse, rat, and human species and is suitable for Flow Cyt, WB, IHC-P, ICC/IF, and IP. This antibody was validated using Western blotting, Immunoprecipitation in this study.
3. Anti-DRP1 antibody [3B5] antibody (Abcam ab56788) reacts with human species and is suitable for IHC-P, IP, and Flow Cyt. This antibody was validated using Western blotting, Immunoprecipitation in this study.
4. Recombinant Anti-Sodium Potassium ATPase antibody [EP1845Y] (Abcam ab76020) antibody reacts with mouse, rat, chinese hamster, and human species and is suitable for ICC/IF, Flow Cyt, WB, and IHC-P. This antibody was validated using immunofluorescence in this study.
5. Recombinant Anti-TOMM20 antibody [EPR15581-54](Abcam ab186735) antibody reacts with mouse, rat, and human species and is suitable for ICC/IF, Flow Cyt, WB, IHC-P, and IHC-Fr.This antibody was validated using immunofluorescence and Western blotting in this study.
6. alpha Tubulin antibody [GT114] (GeneTex GTX628802) reacts with zebrafish, drosophila, mouse, rat, and human species and is suitable for WB, ICC/IF, IHC-P, and IHC-Fr. This antibody was validated using Immunofluorescence in this study.
7. ATP5B antibody [1B10] GeneTex GTX84845) reacts with rat, dog, monkey and human species and is suitable for WB, ICC/IF, IHC-P, and FACS. This antibody was validated using Western blotting in this study.
8. Purified anti-HA.11 Epitope Tag Antibody (BioLegend 901501) is suitable for WB, ICC/IF, IP, and Flow Cyt. This antibody was validated using Western blotting and Immunoprecipitation in this study.
9. Anti-E-Cadherin Antibody, clone 67A4 (Sigma-Aldrich MAB3199Z) reacts with human species and is suitable for WB, ICC/IF, IHC-P, and IHC-Fr. This antibody was validated using Immunofluorescence in this study.
10. Anti-Actin Antibody, clone C4 (Sigma-Aldrich MAB1501) reacts with all animal species and is suitable for ELISA, ICC, IF, IHC, IH(P), and WB This antibody was validated using Western blotting in this study.

Eukaryotic cell lines

Policy information about cell lines and Sex and Gender in Research

Cell line source(s)

Human lung cancer cell line A549 (ATCC Cat# CCL-185) and neuroblastoma cell lines SK-N-DZ (ATCC Cat# CRL-2149, RRID:CVCL_1701), SK-N-BE(2)C (ATCC Cat# CRL-2268, RRID:CVCL_0529), SK-N-SH (ATCC Cat# HTB-11, RRID:CVCL_0531), and SK-N-AS (ATCC Cat# CRL-2137, RRID:CVCL_1700) were purchased from the American Type Tissue Collection (ATCC).

Authentication

All cell lines were authenticated by matching the STR profile to the ATCC public STR Database.

Mycoplasma contamination

All cell lines used in this study were negative for mycoplasma contamination.

Commonly misidentified lines
(See ICLAC register)

No commonly misidentified cell lines were used.

Flow Cytometry

Plots

Confirm that:

☒ The axis labels state the marker and fluorochrome used (e.g. CD4-FITC).

☒ The axis scales are clearly visible. Include numbers along axes only for bottom left plot of group (a 'group' is an analysis of identical markers).

☒ All plots are contour plots with outliers or pseudocolor plots.

☒ A numerical value for number of cells or percentage (with statistics) is provided.

Methodology

Sample preparation	Cells were detached with 1 mM EDTA (J.T. Baker Cat#8991-01) in PBS at room temperature for 5 min after incubation for at least 24 h. DMEM containing 10% fetal bovine serum was used for the inactivation of EDTA and resuspension of the cells. The resuspended cells were centrifuged at 300 × g at 4 °C for 5 min and diluted to a concentration of 1 × 10^6/ml using cold PBS. After fixing with 2% paraformaldehyde at 37 °C in a water bath for 10 min, the non-permeable cells were incubated with the primary antibody against ATP synthase at 4°C overnight. Isotype IgG was loaded under the same conditions as a control. Further hybridization utilizing an Alexa 488-conjugated goat anti-mouse or anti-rabbit IgG was performed at room temperature for 1 h. The labeled cells were washed with cold PBS.
Instrument	BD FACSCanto II instrument (BD Biosciences)
Software	FlowJo 7.6.1 (BD Biosciences)
Cell population abundance	10,000 cells samples was recorded and displayed for each sample.
Gating strategy	FCS/SSC gate was established on bulk of the cells while excluding debris events in the lower left of the panel.

☒ Tick this box to confirm that a figure exemplifying the gating strategy is provided in the Supplementary Information.
